# CircEIF3H-IGF2BP2-HuR scaffold complex promotes TNBC progression via stabilizing HSPD1/RBM8A/G3BP1 mRNA

**DOI:** 10.1038/s41420-022-01055-9

**Published:** 2022-05-14

**Authors:** Xiaojin Song, Bing Chen, Yiran Liang, Yaming Li, Hanwen Zhang, Dianwen Han, Yajie Wang, Fangzhou Ye, Lijuan Wang, Wenjing Zhao, Qifeng Yang

**Affiliations:** 1 Department of Breast Surgery, Qilu Hospital of Shandong University, Jinan, Shandong Province 250012 China; 2 Pathology Tissue Bank, Qilu Hospital of Shandong University, Jinan, Shandong Province 250012 China; 3 Research Institute of Breast Cancer, Shandong University, Jinan, Shandong Province 250012 China

**Keywords:** Breast cancer, Non-coding RNAs

## Abstract

Triple-negative breast cancer (TNBC) is a molecular subtype with an unfavorable prognosis, and metastasis is the main reason for the failure of clinical treatment. However, the expression profile and regulatory function of circRNAs in TNBC progression are not fully understood. Herein, we performed high-throughput RNA-seq in paired breast cancer tissues and adjacent normal tissues and discovered a novel circRNA, circEIF3H, which was upregulated in breast cancer tissues. Large cohort survival analysis confirmed the association between high circEIF3H expression and poor prognosis of TNBC, indicating the vital function of circEIF3H in TNBC progression. Then we conducted both in vitro and in vivo experiments which illustrated that circEIF3H was essential for TNBC proliferation and metastasis. Further experiments showed that circEIF3H did not function as a microRNA sponge as in the most well-established pathway, but as a scaffold for IGF2BP2 and HuR to regulate the mRNA stability of HSPD1, RBM8A, and G3BP1. Our findings provide insight into a novel circRNA, circEIF3H, with significant cancer-promoting function via serving as a scaffold for IGF2BP2/HuR. These results identified circEIF3H as a potential target for developing individualized therapy of TNBC in the approaching future.

## Introduction

Breast cancer is one of the most commonly diagnosed and life-threatening malignant tumors among women worldwide [1]. In United State, there were 284,200 new cases of breast cancer and 44,130 death due to breast cancer in 2021 [2]. Triple-negative breast cancer (TNBC), which is defined as a type of breast cancer with negative expression of estrogen receptor (ER), progesterone receptor (PR), and human epidermal growth factor receptor-2 (HER2), is regarded as an aggressive subtype clinically with the highest recurrence, metastasis, and mortality rate [3]. TNBC is not sensitive to endocrine therapy or molecular targeted therapy [4], therefore, it is critical to further address the molecular mechanisms underlying the development and progression of TNBC, and discover more effective targets for better treatment.

Circular RNAs (circRNAs) have recently been identified as a new class of non-coding RNAs characterized by a covalent closed-loop structure. They are highly stable compared with their linear counterparts because of no 5′ -cap and no 3′ -polyadenylated tail structure. With the development of high-throughput sequencing and bioinformatics analysis, a large amount of circRNAs have been successfully identified and they are abundant, conserved, cell type-specific, or developmental stage-specific, supporting the concept of circRNAs as functional molecules. Studies in recent years reported that circRNAs played important role in broad physiological and pathological processes, especially in oncogenesis and progression of malignant tumors.

The function and underlying mechanism of circRNAs in TNBC have been reported in some previous studies. The most frequently reported mechanism of circRNAs is to trap miRNAs (also known as miRNA sponge) leading to functional loss of target miRNAs and subsequent upregulation of miRNA-targeted genes. For example, CircEPSTI1 promotes TNBC proliferation and apoptosis by upregulating BCL11A expression via binding to miR-4753 and miR-6809 [5], circANKS1B sponges miR-148a-3p and miR-152-3p to increase the expression of USF1 in TNBC [6]. Some circRNAs are capable of binding to proteins and affecting protein interactions. For example, circ-DNMT1 could enhance breast cancer progression by directly binding to p53 and AUF1 and promoting their nuclear translocation [7]. However, more studies are needed to explore and further elaborate the biological functions and regulatory mechanism of circRNAs in TNBC. In this study, we investigated a circular RNA, circEIF3H and we found circEIF3H was overexpressed in TNBC tissues and could promote the TNBC proliferation and metastasis via acting as a scaffold to recruit IGF2BP2 and HuR and subsequently enhancing the stability of downstream mRNA targets (including HSPD1, RBM8A, and G3BP1). Our results suggested that circEIF3H might be a promising therapeutic target to combat TNBC in the future.

## Results

### circEIF3H was correlated with TNBC prognosis

**Fig. 1:**
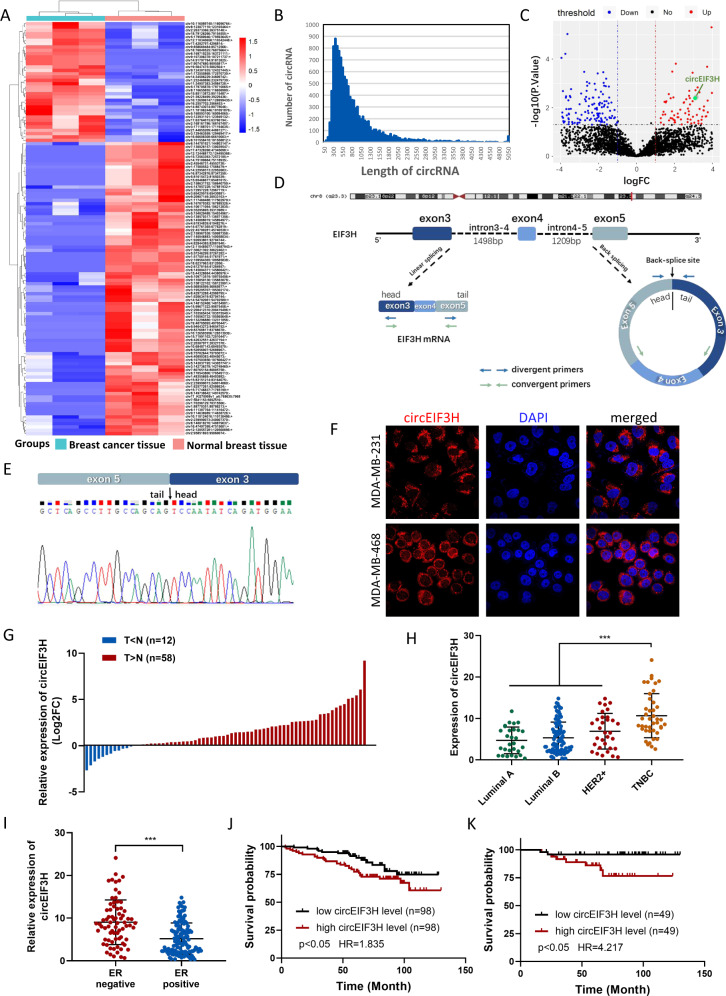
CircEIF3H is upregulated in breast cancer tissues and associated with poor prognosis. **A** Heat maps showing the top differentially expressed circRNAs in three breast cancer samples compared to their adjacent normal tissues. **B** Length distribution of identified circRNAs in three paired breast cancer tissues and adjacent normal tissues. **C** Volcano plots showing the expression profiles of circRNAs. **D** Schematic illustrations showed the genomic loci of circEIF3H. circEIF3H was produced by exons 3 to 5 of the EIF3H gene. **E** Back-splice junction of circEIF3H identified by Sanger sequencing. **F** RNA fluorescence in situ hybridization for circEIF3H. Nuclei were stained with DAPI. **G** The expression level of circEIF3H in 70 pairs of breast cancer tissues and adjacent normal breast tissues. The bar chart presented the log_2_FC. **H**, **I** Expression level of circEIF3H in a different subtype of breast cancer. **J**, **K** Kaplan–Meier analysis showed the association between circEIF3H expression and overall survival of breast cancer patients (*n* = 196) (**J**) or TNBC patients (*n* = 98) (**K**).

To investigate the expression profile of circRNAs in breast cancer, we performed RNA-seq in paired breast cancer tissues and adjacent non-tumor tissues. A total of 81 circRNAs (*P* < 0.01 and fold change >2) were differentially expressed between the breast cancer tissues and paired adjacent normal breast tissues, 28 were upregulated, and 53 were downregulated (Fig. 1A, C). In the present study, we mainly focused on the upregulated circRNAs given that these circRNAs might serve as therapeutic targets or prognostic biomarkers. Among them, circEIF3H, which was one of the prominently upregulated circRNAs in breast cancer tissues with high expression abundance, was chosen for further evaluation.

According to circBase database (http://www.circbase.org/), circEIF3H (hsa_circ_0005231) derives from the translation initiation factor gene *EIF3H* (exon 3–5) on chromosome 8 and ultimately forms the mature circRNA with a length of 418 nt (Fig. 1D). CircEIF3H was highly conservative (Fig. S1A), and the putative junction was validated by Sanger sequencing (Fig. 1E). It was reported that most circRNAs in mammals are processed from internal exons with long flanking introns usually containing complementary sequences, especially ALU repeat elements [8]. Typical long flanking introns with complementary ALU sequence were found in the *EIF3H* gene and they might facilitate the formation of circEIF3H (Fig. S1B, C). Then we performed RNA-FISH with 3′ -Cy3-modification probes and found that circEIF3H was predominantly located in the cytoplasm (Fig. 1F), which was in accordance with the result of the nuclear/cytosol fractionation assay (Fig. S1D). Moreover, circEIF3H was more resistant to RNase R than linear EIF3H (Fig. S1E), and the half-life of circEIF3H was longer than that of linear EIF3H (Fig. S1F). Subsequently, we designed convergent primers to amplify EIF3H mRNA and divergent primers to amplify circEIF3H. Using cDNA and gDNA (genomic DNA) from MDA-MB-231 cell lines as templates, circEIF3H was only amplified by divergent primers in cDNA, and no amplification product was observed in gDNA (Fig. S1G). These results revealed that circEIF3H was an abundant and stable circRNA in breast cancer cells.

To evaluate the association between circEIF3H expression and the clinicopathological features, RT-PCR was performed to detect the circEIF3H expression level in 70 pairs of breast cancer tissues (diagnosed as invasive ductal carcinoma in pathology) and adjacent normal breast tissues. We found that circEIF3H was upregulated in breast cancer in 58 cases whereas downregulated in only 12 cases (Fig. 1G). We also found a higher circEIF3H expression level in ER-negative breast cancer tissues compared to ER-positive tissues (Fig. 1I), and the highest circEIF3H expression in TNBC tissues among four subtypes (luminal A, luminal B, HER2‐enriched, and TNBC) (Fig. 1H), suggesting that circEIF3H may play important role in the progression of TNBC. We further expanded the sample size of breast cancer tissues and a total of 196 breast cancer tissue were included, which was divided into two groups according to their circEIF3H levels (Fig. S1H). High expression of circEIF3H contributed to a poor overall survival in all type of breast cancers (*n* = 196, HR = 1.835, *P* < 0.05) (Fig. 1J) and TNBC subtype (*n* = 98, HR = 4.217, *P* < 0.05) (Fig. 1K). Therefore, we focused our further exploration on the biologic function and mechanism of circEIF3H in TNBC.

### CircEIF3H promotes TNBC proliferation and metastasis

**Fig. 2:**
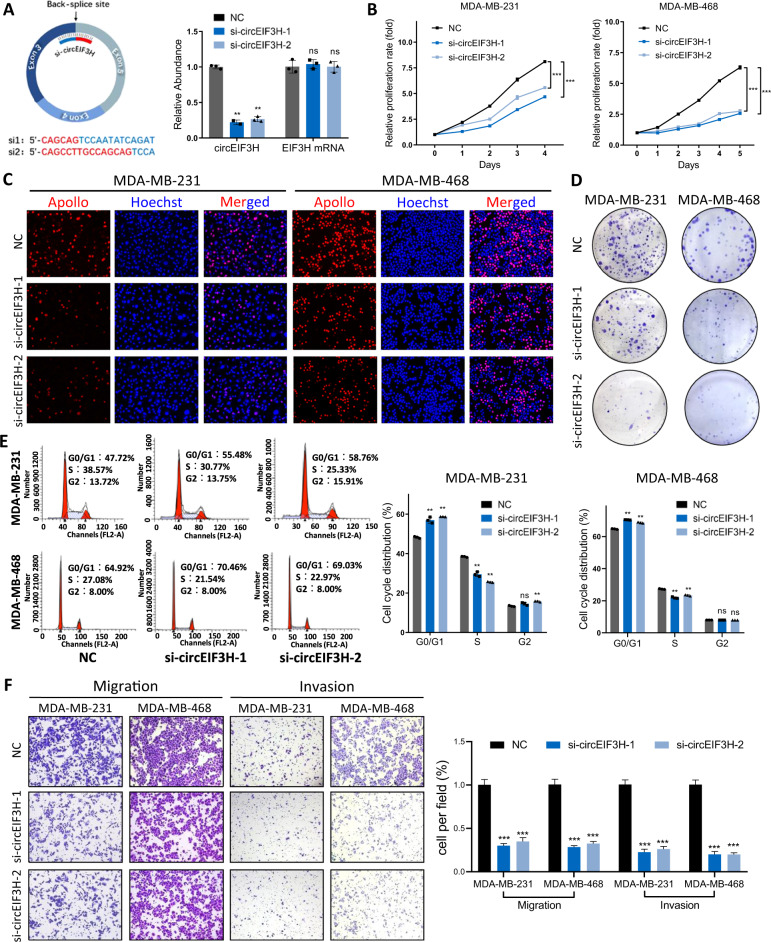
CircEIF3H was essential for TNBC progression. **A** Schematic illustration of siRNAs specific to the back-splicing junction of circEIF3H and the interfering efficacies measured by qRT-PCR. **B**–**D** The effects of circEIF3H knockdown on the proliferation of MDA-MB-231 and MDA-MB-468 cells were examined by MTT (**B**), EdU (**C**), and colony formation assays (**D**). **E** Cell cycle distributions in circEIF3H knockdown cells were presented by flow cytometry. **F** Transwell migration and invasion assays were used to evaluate the motility of MDA-MB-231 and MDA-MB-468 cells transfected with si-NC or si-circEIF3H. The quantitative data were presented as mean ± S.D. Statistical significance was determined by two-sided Student’s *t*-test, **P < 0.01, and ***P < 0.001. Cell experiments were repeated three times.

To investigate the biological functions of circEIF3H in breast cancer cells, we designed interference sequences targeting the back-splice site of circEIF3H. Transfect efficiency was detected by RT-PCR and FISH (Fig. 2A and Fig. S2A), which showed that both two siRNAs (si-circEIF3H-1 and si-circEIF3H-2) could significantly decrease the expression of circEIF3H with no effect on its linear isoform. The results of the MTT and EdU assay showed that circEIF3H knockdown induced striking inhibition of proliferation (Fig. 2B, C). The effects of circEIF3H on cell viability were further mirrored via clonogenic assays and circEIF3H knockdown inhibited clonogenic ability in both MDA-MB-231 and MDA-MB-468 cells (Fig. 2D). Flow cytometry showed that si-circEIF3H led to conspicuous G0/G1 phase arrest and S phase reduction in the cell cycle (Fig. 2E), as well as slightly increased cell apoptosis (Fig. S2C). In consideration, that circEIF3H overexpression is predictive of poor prognosis and metastasis is the leading cause of poor prognosis, we applied a transwell system to test the effects of circEIF3H on cell migration and invasion. CircEIF3H knockdown led to decreased migration and invasion capabilities in breast cancer cells (Fig. 2F). Ectopic expression of circEIF3H was also conducted and the overexpression efficiency was detected by RT-PCT and FISH (Fig. S2A, B). Overexpression of circEIF3H promoted cell growth (Fig. S2D–G), migration, and invasion (Fig. S2H), which was opposite to the effect of circEIF3H knockdown.

### CircEIF3H interacts with IGF2BP2 and HuR as a scaffold complex

Since most circRNAs are reported to work as miRNA sponges in cancer [9–11], we analyzed whether circEIF3H could trap microRNAs. RNA immunoprecipitation (RIP) assay with antibody targeting AGO2, an indicator of circRNA-miRNA interaction, was performed but no significant enrichment of circEIF3H was detected in an anti-AGO2 group than that in the IgG group (Fig. S3). Thus, we suppose that circEIF3H is more likely to work through other mechanisms rather than the miRNA sponge.

**Fig. 3:**
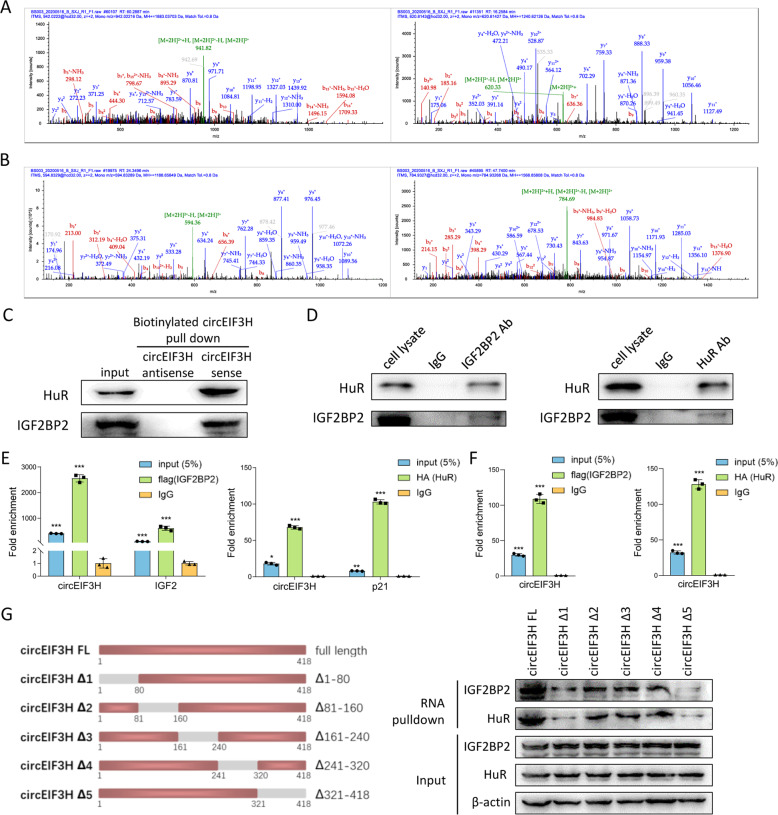
CircEIF3H interacts with IGF2BP2 and HuR as a scaffold. **A** IGF2BP2 protein-specific peptides TVNELQNLTSAEVIVPR and IAPAEGPDVSER were identified by mass spectrometry. **B** HuR protein-specific peptides VLVDQTTGLSR and NVALLSQLYHSPAR were identified by mass spectrometry. **C** Western blot of the proteins from circEIF3H and antisense circEIF3H pull-down assay. **D** Co-IP followed by western blot confirmed the interaction between IGF2BP2 and HuR. **E** RIP assays in 293 T cells showed the association of circEIF3H with Flag-tagged IGF2BP2 and HA-tagged HuR. IgG served as a negative control. **F** RIP assays in MDA-MB-231 cells show the association of circEIF3H with Flag-tagged IGF2BP2 and HA-tagged HuR. **G** Western blot of IGF2BP2 and HuR in samples pulled down by full-length (FL) or truncated circEIF3H (Δ1: 1–80, Δ2: 81–160, Δ3: 161–240, Δ4: 241–320, Δ5: 321–418). The quantitative data were presented as mean ± SD. Statistical significance was determined by two-sided Student’s t-test, *P < 0.05, **P < 0.01, and ***P < 0.001. Cell experiments were repeated three times.

Another generally accepted molecular mechanism of circRNA was circRNA–protein interaction. Therefore, we performed an RNA pull-down assay using a biotin-labeled circEIF3H sequence and the precipitates were subjected to mass spectrometry analysis. Several RNA-binding proteins were identified, including IGF2BP2 and HuR (Fig. 3A, B), which have been reported to interact with each other and promote the stability of their mRNA targets. The result of mass spectrometry was further confirmed by western blot (Fig. 3C). As IGF2BP2 [12] and HuR [13–15] exert cancer-promoting effects in tumorigenesis (Fig. S4A–J), we speculated that circEIF3H might coordinately interact with IGF2BP2/HuR and served as a molecular scaffold. Co-IP (coimmunoprecipitation assays) showed that IGF2BP2 retrieved HuR and HuR retrieved IGF2BP2 (Fig. 3D), which suggested that IGF2BP2 and HuR physically bound with each other. RIP assay (Fig. 3E) in 293 T cells showed that circEIF3H was enriched in RNA binding to Flag-tagged IGF2BP2 compared with a control group (IGF2 mRNA as positive control) [11], and circEIF3H was enriched in RNA binding to HA-tagged HuR (P21 mRNA as positive control) [16]. Besides, the RIP assay in MDA-MB-231 showed a similar result (Fig. 3F). We then constructed a series of circEIF3H truncations to map its binding fragment with IGF2BP2 and HuR. Deletion-mapping analyses identified that 1-80nt and 321-418 nt in circEIF3H were required for its association with IGF2BP2/HuR (Fig. 3G). It’s worth noting that this region was identically the unique back-splicing site on circEIF3H that did not exist in any linear mRNAs or other circRNAs derived from the same host gene (*EIF3H*). Taken together, these results indicate that circEIF3H directly binds to IGF2BP2/HuR and serves as a scaffold to induce the formation of the circEIF3H-IGF2BP2-HuR complex.

### CircEIF3H-IGF2BP2-HuR scaffold complex stabilizes HSPD1, RBM8A, and G3BP1 mRNA

**Fig. 4:**
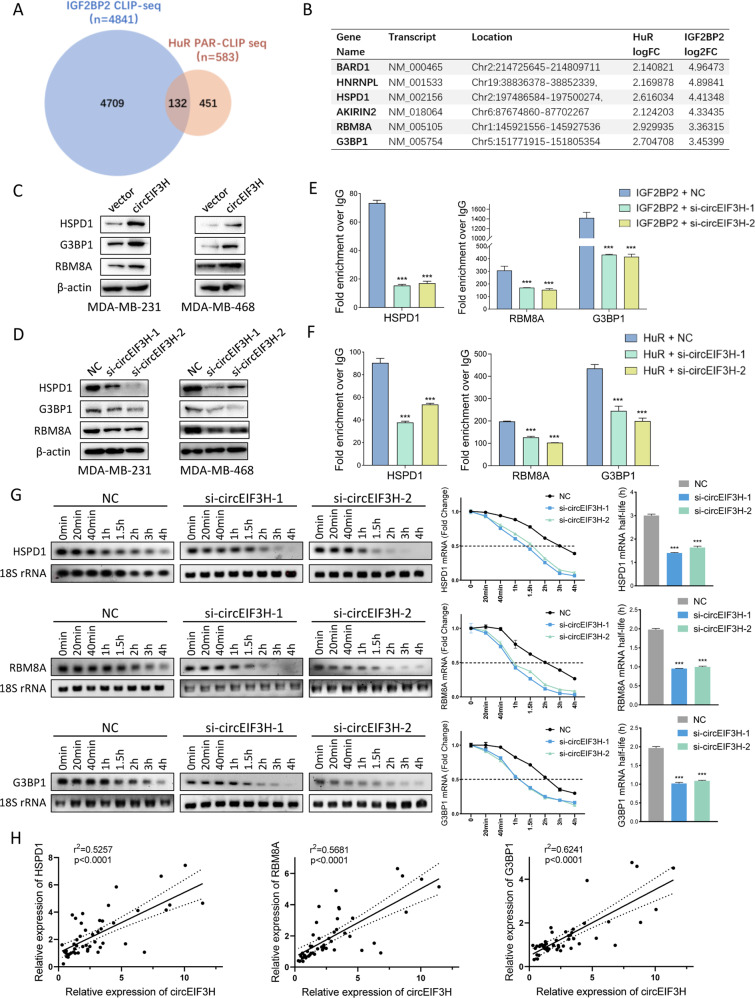
CircEIF3H-IGF2BP2/HuR complex stabilized HSPD1/RBM8A/G3BP1 mRNA. **A** Venn diagram shows the RNAs interacted with IGF2BP2 (4709), HuR (451), or both (132). **B** Top transcripts interacted with both IGF2BP2 and HuR ware listed. **C**, **D** Expression levels of HSPD1/RBM8A/G3BP1 after circEIF3H knockdown (**C**) or overexpression (**D**) were detected by Western blot. **E**, **F** RIP assays showed the enrichment of HSPD1/RBM8A/G3BP1 with Flag-tagged IGF2BP2 (**E**) or HuR (**F**). **G** mRNA stability of HSPD1/RBM8A/G3BP1 was analyzed after si-circEIF3H transfection. **H** Correlation analysis of circEIF3H and HSPD1/RBM8A/G3BP1 expression analyzed by qRT-PCR in breast cancer tissues (*n* = 49). The quantitative data were presented as mean ± SD. Statistical significance was determined by a two-sided Student’s *t*-test, ***P < 0.001. Cell experiments were repeated three times.

As both IGF2BP2 and HuR is essential for mRNA stability [10–12, 17], we then wondered if the circEIF3H-IGF2BP2-HuR complex stabilizes certain unknown downstream targets. IGF2BP2 enhanced-CLIP seq data (GSE90639) and HuR enhanced-PAR-CLIP seq data (GSE29780) were analyzed and six genes were selected from the intersection (Fig. 4A, B). Through further WB validation, we confirmed that HSPD1, RBM8A, and G3BP1 were the potential targets of IGF2BP2 and HuR because the expression level of circEIF3H was positively correlated to them (Fig. 4C, D). This positive correlation between circEIF3H and HSPD1/RBM8A/G3BP1 was also detected in 3 different TNBC cell lines (MDA-MB-231, MDA-MB-468, and MDA-MB-453), which further confirmed our result (Fig. S5A). RIP assay was performed to verify the interaction between IGF2BP2/HuR and HSPD1/RBM8A/G3BP1, and the results showed that HSPD1/RBM8A/G3BP1 were significantly enriched in both IGF2BP2 (Fig. 4E) and HuR (Fig. 4F) precipitates. To further confirm that circEIF3H, IGF2BP2, and HuR served as a complex to bind their target mRNAs, we performed an anti-IGF2BP2 RIP assay with circEIF3H overexpression or HuR-si and found that more target mRNAs (HSPD1/RBM8A/G3BP1) enriched in IGF2BP2 precipitates with circEIF3H overexpression, whereas less target mRNAs enriched with HuR-si transfection (Fig. S5B). Similar results were obtained when we performed an anti-HuR RIP assay with circEIF3H overexpression or IGF2BP2-si (Fig. S5C). Then we explored whether the circEIF3H-IGF2BP2-HuR complex could stabilize their target mRNAs to prolong mRNA half-life. As shown in Fig. 4G, the half-life of HSPD1, RBM8A, and G3BP1 were decreased with circEIF3H knockdown, which was in accordance with our hypothesis. We further examined the correlation between circEIF3H and HSPD1/RBM8A/G3BP1 by qRT-PCT in 49 human breast cancer tissues and confirmed the positive correlation between circEIF3H and HSPD1/RBM8A/G3BP1 respectively (Fig. 4H). In summary, these findings demonstrated that circEIF3H is bound to IGF2BP2 and HuR and served as a complex to stabilize their target mRNAs (HSPD1, RBM8A, and G3BP1).

### HSPD1/RBM8A/G3BP1 promotes TNBC progression

Accumulating evidence showed that HSPD1, RBM8A, and G3BP1 played a key role in tumor progression in several cancers; however, its effect on breast cancer proliferation and metastasis remains unclear. HSPD1/RBM8A/G3BP1 siRNAs were transfected into TNBC cells, and the transfection efficiency was determined by RT-PCR (Fig. S6A). MTT assay showed that knockdown of HSPD1/RBM8A/G3BP1 respectively impaired TNBC cell proliferation (Fig. S6B), in accordance with less Edu incorporation in HSPD1/RBM8A/G3BP1 silenced cells (Fig. S6C). Besides, knockdown of HSPD1/RBM8A/G3BP1 significantly suppressed colony-forming ability (Fig. S6D). Alterations in metastatic capacity were further evaluated, and a prominently decrease in cell migration and invasion was observed in HSPD1/RBM8A/G3BP1 silencing cells (Fig. S6E).

To investigate whether HSPD1/RBM8A/G3BP1 were involved in breast cancer progression clinically, the BCIP database (http://www.omicsnet.org/bcancer/) was used to analyze the relationship between HSPD1/RBM8A/G3BP1 expression and clinicopathological characteristics of breast cancer patients. We found that all these three genes were highly expressed in breast cancer tissues compared to adjacent normal breast tissues and in TNBC tissues compared to non-TNBC tissues. Higher expression levels were also observed in breast cancer patients with a higher histological grade as well as higher pathological stage (Fig. S7A). Furthermore, Kaplan–Meier survival analysis showed that breast cancer patients with high HSPD1, RBM8A, or G3BP1 expression respectively had significantly worse distant metastasis-free survival (DMFS) in the TCGA Cohort (Fig. S7B). Taken together, these findings strongly suggested that HSPD1/RBM8A/G3BP1 could promote breast cancer progression and were correlated with poor prognosis.

### HSPD1/RBM8A/G3BP1 silence reverses the cancer-promoting effect of circEIF3H

**Fig. 5:**
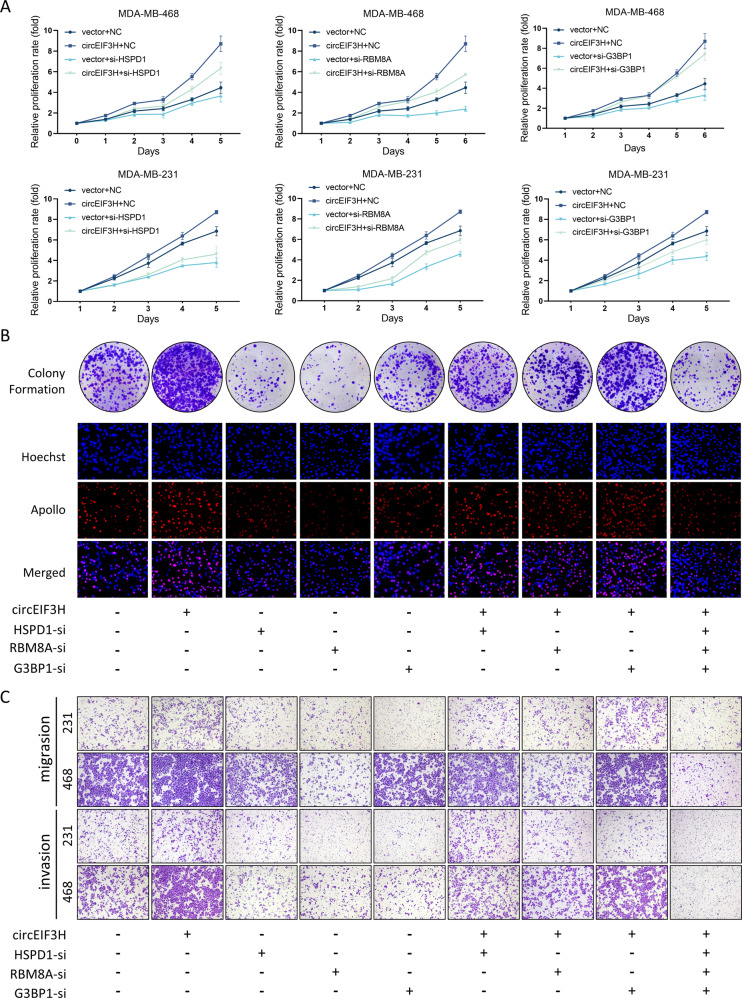
HSPD1/RBM8A/G3BP1 silence rescues the cancer-promoting effect of circEIF3H. **A**–**C** TNBC cells transfected with pLCDH-ciR, circEIF3H, si-NC, or si- HSPD1/RBM8A/G3BP1 alone or simultaneously. Then the abilities of cell proliferation, colony formation, migration, and invasion were detected respectively, assessed by MTT (**A**), colony formation assay and EdU assay (**B**), transwell migration and matrigel invasion assay (**C**). The quantitative data were presented as mean ± SD. Statistical significance was determined by two-sided Student’s *t*-test, **P < 0.01, ***P < 0.001. Cell experiments were repeated three times.

To explore whether circEIF3H enhanced proliferation, migration, and invasion in TNBC by interacting with IGF2BP2/HuR and stabilizing HSPD1/RBM8A/G3BP1 mRNA, rescue experiments were performed. TNBC cells were cotransfected with circEIF3H overexpression plasmid and HSPD1/RBM8A/G3BP1 siRNAs respectively. CircEIF3H overexpression significantly promoted TNBC cell proliferation (Fig. 5A), Edu incorporation and colony formation (Fig. 5B and Fig. S8A) in TNBC cells, and HSPD1/RBM8A/G3BP1 knockdown attenuated the cancer-promoting effect induced by the circEIF3H overexpression. In addition, transwell migration and invasion assays (Fig. 5C and Fig. S8B, C) showed that circEIF3H overexpression markedly ameliorated the tumor-suppressive effect of HSPD1/RBM8A/G3BP1 silencing. These results indicated that the TNBC-promoting effect of circEIF3H depended on the upregulation of HSPD1/RBM8A/G3BP1 expression.

### CircEIF3H promotes tumor progression in mice xenografts and lung metastatic model

**Fig. 6:**
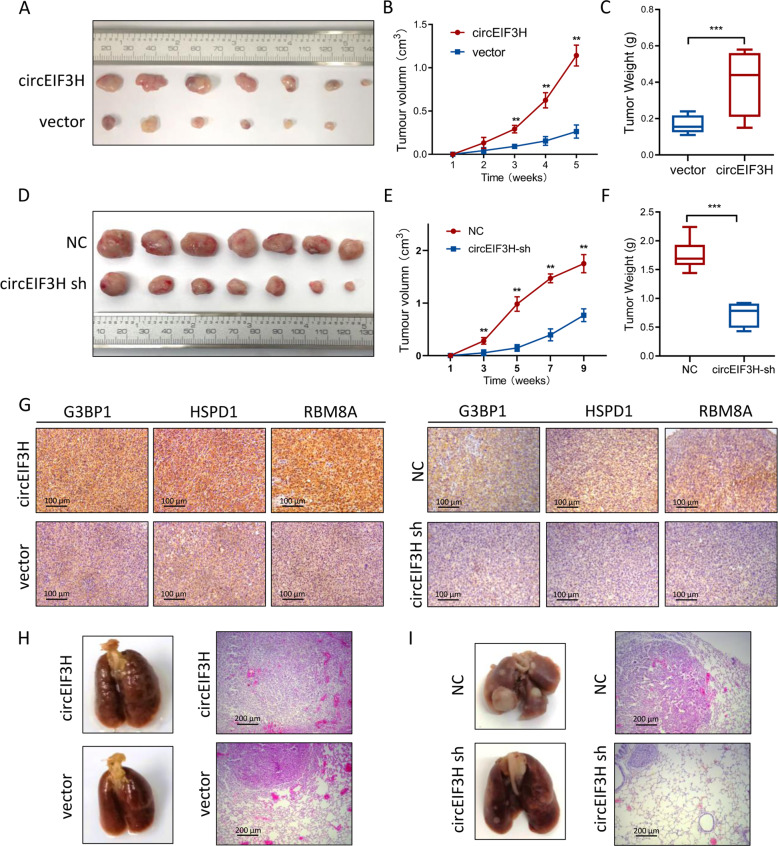
CircEIF3H promotes tumor growth and metastasis in vivo. **A** MDA-MB-231 cells were stably transfected with the circEIF3H-overexpressing vector or control vector and injected subcutaneously into nude mice. CircEIF3H overexpression promoted tumor growth compared with the vector group. **B**, **C** Tumor growth rate (**B**) and tumor weight (**C**) of circEIF3H-overexpressing group and vector group were presented as mean ± SD; circEIF3H-overexpressing group *n* = 7; vector group *n* = 6; **P < 0.01, ***P < 0.001. **D** MDA-MB-231 cells were stably transfected with the circEIF3H-sh vector or control vector and injected subcutaneously into nude mice. CircEIF3H-sh inhibited tumor growth compared with the vector group. **E**, **F** Tumor growth rate (**E**) and weight (**F**) were significantly decreased in the circEIF3H-sh group. Tumor growth rate (**E**) and tumor weight (**F**) of circEIF3H-sh group and NC group were presented as mean ± SD; *n* = 7; **P < 0.01, ***P < 0.001. **G** IHC staining analysis of the expression of HSPD1, RBM8A, and G3BP1 in tumors. Scare bar = 100 μm. **H**, **I** The macroscopic view of lung metastasis nodules and H&E-stained lung section images (Scare bar = 200 μm) after tail vein injection of MDA-MB-231 cells with circEIF3H-overexpression or circEIF3H-sh (*n* = 5). Statistical significance was determined by a two-sided Student’s *t*-test.

To study the role of circEIF3H in vivo, we injected circEIF3H-overexpressing (or circEIF3H-sh) MDA-MB-231 cells and control cells into the flank of BALB/c mice. After 6 weeks (for circEIF3H overexpression) or 10 weeks (for ciecEIF3H-sh), the mice were sacrificed. The data consolidated the biological function of circEIF3H in vitro, manifested by a higher rate of tumor growth and heavier tumor weight in the circEIF3H-overexpressing group (Fig. 6A–C), and a lower rate of tumor growth and lighter tumor weight in the circEIF3H-sh group (Fig. 6D–F). Higher expression levels of HSPD1/RBM8A/G3BP1 were detected in circEIF3H-overexpressing group compared with the control group via immunohistochemistry, whereas lower expressions were detected in the circEIF3H-sh group (Fig. 6G). As lung metastasis has been recognized as an indicator of tumor invasiveness, we then injected circEIF3H-overexpressing (or circEIF3H-sh) MDA-MB-231 cells into the tail vein of nude mice to establish a lung metastatic model, and the mice were sacrificed after 8 weeks (for circEIF3H overexpression) or 12 weeks (for circEIF3H-sh). In coherence to in vitro results, more metastatic nodules with bigger volume were found in the circEIF3H overexpression group (Fig. 6H), whereas less nodules with smaller volume were found in the circEIF3H-sh group (Fig. 6I). H&E staining was performed to pathologically confirm the metastatic nodules in the lungs (Fig. 6H, I). In summary, these data indicated that circEIF3H strongly promoted TNBC proliferation and metastasis in vivo.

## Discussion

CircRNA is a type of non-coding RNA that has been identified in the past several years and has drawn more and more attention from scientists worldwide. They are formed by covalent linkage of the ends of a single RNA molecule during the process of RNA splicing and characterized by its high stability, high conservation, cell type-specificity, and developmental stage-specificity. With the development of high-throughput sequencing technologies, accumulating evidence have proved the function of circRNAs in both physiological and pathological processes. It has been reported that circRNAs played important roles in carcinogenesis, progression, and metastasis of multiple cancers, such as hepatocellular carcinoma [18, 19], colorectal cancer [20], bladder cancer [21], and TNBC. However, most of the reported regulatory mechanism of circRNAs in TNBC was microRNA sponging, which means that circRNAs could trap miRNAs leading to functional loss of target miRNAs and subsequent upregulation of miRNA-targeted genes. However, more studies are needed to further elaborate on the biological functions and other possible regulatory mechanisms of circRNAs in TNBC.

In the present study, we discovered a new circRNA, circEIF3H, through high-throughput RNA sequencing in paired breast cancer tissues and adjacent non-tumor tissues. CircEIF3H was highly expressed in TNBC tissues and was associated with poor prognosis in breast cancer patients, implicating circEIF3H as a valuable marker for prognosis judgment. However, there is a limitation in our present study that the analysis of the association between circEIF3H expression and patient clinicopathological characteristics was not performed. Thus, we cannot determine whether circEIF3H would function as a clinically useful biomarker for the evaluation of TNBC progression. In future studies, more TNBC tissues are needed to assess the clinical predictive value of circEIF3H. In addition, the levels of serum circEIF3H expression could be analyzed in further studies to assess its ability as a liquid biopsy biomarker.

Furthermore, biological functional experiments were conducted in our present study and revealed that circEIF3H was essential for TNBC proliferation and metastasis, in consistent with the results of experiments in mice bearing xenografts and lung metastatic model. The competing endogenous RNAs (ceRNAs) mechanism is the most classic and well-established theory explaining the biological function of circRNAs in recent years. Thus, we performed an anti-AGO2 RIP assay and found that circEIF3H was not significantly enriched in AGO2 precipitation, suggesting that circEIF3H may promote TNBC progression through other mechanisms.

Studies in recent years showed that circRNAs could interact with different proteins to form a specific circRNA–protein complex that subsequently influences the modes of action of associated proteins. For example, circ-FOXO3, a highly expressed circRNA in the mammalian heart, could promote cardiac senescence by interacting with senescence-related proteins ID1 and E2F1 and tumor-related proteins HIF1α and FAK [22]. It was also observed that circ-Foxo3 could bind to p21 and CDK2 and induce cell cycle arrest via the formation of circ-Foxo3-p21-CDK2 ternary complex [23]. Therefore, we considered whether circEIF3H could bind to some specific protein and modulate the interactions and functions of these proteins. We found that circEIF3H could bind to IGF2BP2 and HuR proteins, which were confirmed through RNA pull-down assay followed by mass spectrometry analysis and RIP assay. As both IGF2BP2 and HuR were previously reported as RNA-binding proteins for mRNA stabilization, we analyzed CLIP seq for both IGF2BP2 and HuR and found three target genes (HSPD1, RBM8A, and G3BP1) among the overlapped parts. HSPD1, also called HSP60, is essential for the folding and assembly of newly imported proteins in mitochondria. In addition, HSP60 is actively secreted by cancer cells and plays a role in transformation, angiogenesis, and metastasis [24, 25]. RBM8A was reported to predict poor prognosis and promote tumor progression in hepatocellular carcinoma [26], but its function in breast cancer remains unknown. G3BP1 promotes tumor cell proliferation and metastasis and inhibits apoptosis through Ras, TGF-β/Smad, Src/FAK, and p53 signaling pathways [27]. In breast cancer, G3BP1 supported cell proliferation via increasing PMP22 expression [28]. Taken together, all these three genes exerted a potent cancer-promoting effect. Here, we found that circEIF3H can function as a scaffold for IGF2BP2 and HuR to regulate the expression of HSPD1, RBM8A, and G3BP1 by enhancing their mRNA stability. This hypothesis was verified with three lines of solid experimental evidence: (1) circEIF3H physically interacts with IGF2BP2 and HuR; (2) circEIF3H overexpression promoted the recruitment of IGF2BP2 and HuR to their target genes; (3) the half-life of HSPD1, RBM8A, and G3BP1 mRNAs were decreased with circEIF3H knockdown.

In summary, we performed high-throughput RNA sequencing in paired breast cancer tissues and adjacent normal breast tissues and identified a circular RNA, circEIF3H, which was highly expressed in breast cancer tissues, especially in TNBC tissues. Both in vitro and in vivo experiments illustrated the significant cancer-promoting functions of circEIF3H. Clinically, circEIF3H contributed to poor prognosis in breast cancer patients, indicating the great clinical significance of circEIF3H. Further studies revealed that circEIF3H served as a scaffold for IGF2BP2/HuR and subsequently promoted the stabilization of their target mRNAs (HSPD1/RBM8A/G3BP1). Our findings provide insight into circEIF3H biologic function and uncovered its scaffold mechanism, therefore providing a theoretical basis for circEIF3H serving as a novel prognostic biomarker and a new target for developing tailored therapy of TNBC in the approaching future.

## Materials and methods

### Cell culture

Human breast cancer cells (MDA-MB-231, MDA-MB-468, and MDA-MB-453) and 293 T were purchased from the American Type Culture Collection (Manassas, VA, USA). The cell lines were characterized by Genetic Testing Biotechnology Corporation (Suzhou, China) using short tandem repeat markers. Cells were routinely cultured in Dulbecco’s Modified Eagle medium (Gibco-BRL, Rockville, IN, USA) with 10% heat-inactivated fetal bovine serum (Hyclone), 100 U/ml of penicillin, and 100 μg/ml of streptomycin. Cells were maintained at 37 °C in a humidified atmosphere with 5% CO_2_.

### Tissue specimens and ethics statement

This study has been approved by the Ethical Committee on Scientific Research of Shandong University Qilu Hospital. Written informed consent was obtained from the patients before the study began.

Human breast cancer and adjacent normal tissues were collected from patients receiving surgery with informed consent at Qilu Hospital from July 2008 to January 2015. All the patients were followed up on a regular basis, overall survival (OS) time was determined from the date of surgery to the date of death or the date of the last follow-up visit for survivors.

### RNA sequencing

Three paired breast cancer tissues and adjacent normal breast tissues were used for RNA sequencing analysis. Total RNAs were extracted with Trizol (Invitrogen, Carlsbad, CA, USA). RNA integrity was analyzed with Agilent 4200 Bioanalyzer (Agilent Technologies, Santa Clara, CA, USA). RNA concentration was tested by Qubit RNA Assay Kit in Qubit Fluorometer (Invitrogen, Carlsbad, CA, USA). Total RNA samples that meet the following requirements were used in subsequent experiments: RNA integrity number (RIN) ≥7.0.

Sequencing libraries were generated and sequenced by Gminix (Shanghai, China). A total amount of 4 μg RNA per sample was used. Briefly, total RNAs were subjected to ribosomal RNA (rRNA) removal, DNaseI digestion, and RNase R digestion. The VAHTSTM Total RNA-seq (H/M/R) Library Prep Kit for Illumina^®^ (Vazyme #NR603) was used to construct the libraries for sequencing according to the manufacturer’s protocol. The RNA was then fragmented and the first-strand cDNA was synthesized from the RNA fragments by reverse transcriptase and random hexamer primers, and the second-strand cDNA was synthesized in Second Strand Synthesis Reaction Buffer with dUTP Mix (10x). Adapters were ligated at 3′ and 5′ ends using T4 ligase, and RT‐PCR was performed for amplification. The PCR products were further purified and were used for sequencing. Procedures were performed as described in detail on the website of Gminix (http://www.gminix.com/).

### RNA-FISH and subcellular fractionation

Specific probes to hsa_circ_0005231 (circEIF3H) (ATATTGGACTGCTGGCAAGGCTG) was prepared (GenePharma, China). Cell nuclei were counterstained with DAPI. Finally, images were obtained on a Zeiss LSM 700 confocal microscope (Carl Zeiss, Oberkochen, Germany). Nuclear and cytoplasmic separation was performed using the PARIS Kit (Life Technologies, USA) according to the manufacturer’s instructions.

### RNA extraction and qRT-PCR

Total RNA was prepared from breast cancer cells using the TRIzol reagent (Invitrogen, CA, USA) according to the manufacturer’s instructions. Then cDNA was synthesized using PrimeScript reverse transcriptase (RT) reagent kit (TaKaRa, Shiga, Japan). qRT-PCR was carried out using the SYBR green PCR mix (Takara) and β-actin was used as the endogenous control. The primers were listed in Supplemental Table 1.

### Actinomycin D and RNase R treatment

Transcription was prevented by the addition of 2 μg/ml Actinomycin D (Sigma-Aldrich, USA) for the indicated time. Total RNA (2 μg) was incubated for 30 min at 37 °C with 3 U/μg of RNase R. After treatment with Actinomycin D and RNase R, the RNA expression levels of circEIF3H and EIF3H mRNA were detected by qRT-PCR using the SYBR green PCR mix (Takara).

### Plasmid construction and cell transfection

pLCDH-ciR plasmid was brought from Geneseed (Guangzhou, China) and was used to overexpress circEIF3H. According to the instruction, there is a stuffer sequence of 139 bp between two cleavage sites of restriction enzyme (EcoRI and BamHI) in this plasmid. This original plasmid brought from Geneseed was used in our study as a negative control. The full-length cDNA of circEIF3H (exon 3 to exon 5 of EIF3H gene) was amplified and then cloned into pLCDH-ciR between two cleavage sites of restriction enzyme (EcoRI and BamHI). There is a front and back circular frame on pLCDH-ciR vector. The result of vector construction was verified by direct sequencing. For the transient transfection, the overexpression plasmid and the corresponding control plasmid were transfected into breast cancer cell lines using lipofectamine 2000 (Invitrogen) according to the manufacturer’s instructions. For the lentivirus package, HEK-293 T cells were transfected with the core plasmid pLCDH-ciR (Geneseed Biotech, Guangzhou, China), with the psPAX2 packaging plasmid and pMD2.G envelope plasmid for 48 h to obtain the lentivirus supernatant. The transfected cells were screened with puromycin for 4 weeks to establish circEIF3H overexpression and control cell lines. The pEnter-IGF2BP2 (Vigene Bioscience) was commercially obtained. The full-length IGF2BP2 cDNA was cloned into pENTER (Invitrogen, USA). The full-length HuR cDNA was cloned into pCMV-N-HA (Beyotime, China) to conduct a HuR overexpression plasmid. For the knockdown experiments, the small interfering RNA (siRNA) target sequences (GenePharma, China) was transfected using Lipofectamine 2000 (Invitrogen). The sequences of siRNAs were listed in Supplemental Table 2. To obtain stable circEIF3H-sh cells, short hairpin RNAs (shRNAs) against circEIF3H was constructed, which inserted siRNA into the pSuper.puro vector. Puromycin was used for 3–4 weeks to select stably transfected cell lines. Furthermore, clonal selection and qRT-PCR analysis were performed to confirm the circEIF3H-sh expression in individual clones.

### Antibodies

The IGF2BP2 antibody (Proteintech, Cat. 11601-1-AP), HuR antibody (Proteintech, Cat. 11910-1-AP), HSPD1 antibody (Proteintech, Cat. 15282-1-AP), RBM8A antibody (Proteintech, Cat. 14958-1-AP), G3BP1 antibody (Proteintech, Cat. 13057-2-AP), Flag antibody (Thermo, Cat. PA1-984B), poly-HA antibody (Sigma, Cat. H6908) were applied in the study.

### EDU incorporation assay

Cells were seeded in 96-well plates at 2 × 10^4^ per well 24 h after transient transfection (in the logarithmic phase of growth) and incubated at 37 °C in a humidified 5% CO_2_ atmosphere for 12 h. Then cell-Light™ EDU DNA Cell Proliferation (EdU: 5′Ethynyl-2′-deoxyuridine) kit (Ribobio, Guangzhou, China) was used to monitor the proliferation of breast cancer cells according to the manufacturer’s instructions. A fluorescence microscope (Olympus, Tokyo, Japan) was used to count the cells in proliferating phase (exciting bright red spots) and the total cells (blue ones). Each experiment was performed in triplicate.

### 3-(4,5-Dimethylthiazol-2-yl)-2,5-diphenyltetrazolium bromide (MTT) assay

The transfected cells were seeded into 96-well plates at a density of 1500 cells per well. After incubation, 20 μl of 5 mg/ml MTT was added to each well and incubated for another 4 h. Then, the supernatants were carefully removed, and 100 μl DMSO was added to each well. The proliferation curves were determined by calculating the relative value of absorbance measured at 570 nm on a microplate reader (Bio-Rad, USA).

### Cell cycle assay

Cell cycle assays were performed 48 h after transfection. The transfected breast cancer cells were stained with 500 μl cell cycle staining buffer (MultiSciences (Lianke), Hangzhou, Zhejiang, China) for 30 min in a dark place and then measured by flow cytometry (Becton Dickinson, Franklin Lakes, NJ, USA). The data were analyzed with ModFit LT V4.0 software.

### Colony formation assay

Transfected cells were counted and seeded at 500 cells per 6 cm plate. After 16–20 days, cell colonies were washed with PBS, fixed with methanol for 15 min, and stained with crystal violet for 20 min. The colonies were imaged and counted.

### Cell migration and invasion assay

For migration assays, 8 × 10^4^ cells were plated in 200 μl of serum-free medium to a Boyden chamber (BD Biosciences, NewJersey, USA) in the insert of a 24-well plate after transfection. Medium containing 20% FBS was added to the bottom chamber. After incubation for 24 h (for MDA-MB-231) or 36 h (for MDA-MB-468), cells in lower filters were fixed in methanol, and stained in crystal violet (Sigma, MO, USA), and counted under a microscope. The invasion assay was performed in the same way as the migration assay except that the membrane was coated with matrigel (BD Biosciences, Bedford, MA, USA). Each experiment was performed in triplicate.

### Protein isolation and western blot

Cells were collected and lysed with Western and IP lysis buffer (Beyotime) with protease inhibitors. Equal amounts of proteins were loaded on SDS-PAGE gels and then transferred to a PVDF membrane (Millipore). After blocking with 5% nonfat milk, the membrane was incubated overnight at 4 °C with the primary antibody. Blots were washed and incubated with horseradish peroxidase-coupled secondary antibody (Millipore) for 2 h. Signal was detected with enhanced chemiluminescence (Millipore).

### In vitro transcription

The DNA template used for in vitro synthetization of biotinylated circEIF3H was generated by PCR amplification. The forward primer contained the T7 RNA polymerase promoter sequence to allow for subsequent in vitro transcription. PCR products were purified using the DNA Gel Extraction Kit (Tiangen), and in vitro transcription was performed using the MEGAscript^®^ T7 Kit (Thermo Fisher Scientific) according to the manufacturer’s instructions.

### Biotin RNA pull-down assay

CircEIF3H was labeled with 3′ biotin using Pierce™ RNA 3′ End Desthiobiotinylation Kit (Thermo Fisher Scientific). MDA-MB-231 cells were lysed with lysis buffer and incubated with biotin-labeled circEIF3H. The cell lysates were incubated with streptavidin-coated magnetic beads to pull down the biotin-labeled RNA complex using Pierce™ Magnetic RNA-Protein Pull-Down Kit (Thermo Fisher Scientific). Then the beads were collected and RNA-associated proteins were eluted and resolved by mass spectrometry and western blot analysis.

### Coimmunoprecipitation (Co-IP)

Cells were harvested at 48 h post-transfection and lysed in NP40 lysis buffer supplemented with protease inhibitor cocktail (Roche) for 30 min on ice. Indicated primary antibody and control IgG were added to the lysate separately and incubated on a rotator at 4 °C for 1 h. Afterward, 40 μl protein A/G agarose beads (Santa Cruz) were added and incubated at 4 °C overnight on a rotator. Immunoprecipitates were examined using the indicated primary antibodies in the same way as the immunoblotting assay.

### RNA immunoprecipitation

RNA immunoprecipitation (RIP) assays were performed using the Magna RIP RNA-Binding Protein Immunoprecipitation Kit (Millipore, Bedford, MA) according to the manufacturer’s instructions. Briefly, cells were lysed in a complete RIP lysis buffer containing a protease inhibitor cocktail and an RNase inhibitor. Flag/HA antibodies were used. RNA was purified from the RNA–protein complexes that bound to the beads and then was analyzed by real-time RT-PCR. To demonstrate that the detected RNA signals specifically bind to Flag-IGF2BP2 or HA-HuR, total RNA (input controls) and normal rabbit IgG controls were simultaneously assayed. The gene-specific primers used for detecting IGF2BP2/HuR binding genes are displayed in Supplemental Table 1.

### Immunofluorescence staining

For immunofluorescence, the transfected cells were seeded on glass coverslips for 24 h and then incubated with primary antibodies at 4 °C overnight. Cell nuclei were counterstained with 2 μg/ml Hoechst for 5 min. Images were captured with a microscope using 20× objectives.

### Tumor model and metastasis assay in vivo

Female BALB/c nude mice (4-week-old) were randomly divided into two groups, with seven mice in each group. Then MDA-MB-231 cells stably transfected with circEIF3H overexpression plasmids or circEIF3H-sh plasmids were injected subcutaneously into the upper back of mice (5 × 10^6^, 100 μl) randomly. Tumor growth was monitored weekly and calculated as 1⁄2 LD^2^ (L, longitudinal diameter; D, latitudinal diameter). When mice were sacrificed 6 weeks (for circEIF3H overexpression) or 10 weeks (for circEIF3H-sh) later, tumors were harvested, weighed, and stained. About 1 × 10^5^ cells were injected into the lateral tail veins of 4-week-old BALB/c nude mice randomly to produce lung metastasis. Eight weeks (for circEIF3H overexpression) or 12 weeks (for circEIF3H-sh) later, lungs were collected and stained with HE. No blinding was performed for the animal experiments. These experiments were approved by the Animal Care and Use Committee of Shandong University.

### Immunohistochemistry (IHC)

The paraffin-embedded sections were dewaxed in xylene and rehydrated in alcohol. Endogenous peroxidase was blocked by 3% H_2_O_2_, and microwave heating was performed for antigen retrieval. After blocking nonspecific antigen binding with 5% BSA at 37 °C for 1 h, the sections were incubated with a specific primary antibody against HSPD1, RBM8A, or G3BP1 at 4°C overnight. After incubating with the corresponding secondary antibodies at 37 °C for 1 h, the sections were stained with diaminobenzidine and counterstained with hematoxylin. Representative images were taken using an Olympus light microscope.

### Kaplan–Meier plotter tool analysis

The Kaplan–Meier plotter tool (http://kmplot.com/analysis/) was used to determine the association between HSPD1, RBM8A, G3BP1, and the prognosis of breast cancer patients.

### Statistical analysis

The software SPSS V22.0 was used for statistical analysis. Normal distribution test and test of homogeneity of variances were analyzed. The data in statistical tests are conformed to a normal distribution and the variance is similar. Statistical significance was assessed using the Student’s *t*-test or one-way analysis of variance (ANOVA) followed by post hoc tests. Kaplan–Meier plots were tested by log-rank tests. Correlations between circEIF3H and HSPD1/RBM8A/G3BP1 were analyzed by Spearman rank correlation. Statistical differences were indicated as *P < 0.05, **P < 0.01, and ***P < 0.001. All quantitative data presented were the mean ± SD from at least three independent experiments.

## Supplementary information

Supplementary figures

Supplementary table

Original Data File

aj-checklist

## Data Availability

The datasets supporting the conclusions of this article are available from the corresponding author on reasonable request.
