# Supplementary material for: CircEIF3H-IGF2BP2-HuR scaffold complex promotes TNBC progression via stabilizing HSPD1/RBM8A/G3BP1 mRNA
Source: Cell Death Discov. 2022 May 14;8:261. doi: 10.1038/s41420-022-01055-9 (PMC9107465; doi:10.1038/s41420-022-01055-9)
Supplement: Supplementary file 1 — Supplementary figures [file 41420_2022_1055_MOESM1_ESM.docx]

**Supplementary Figures and Figure legends**


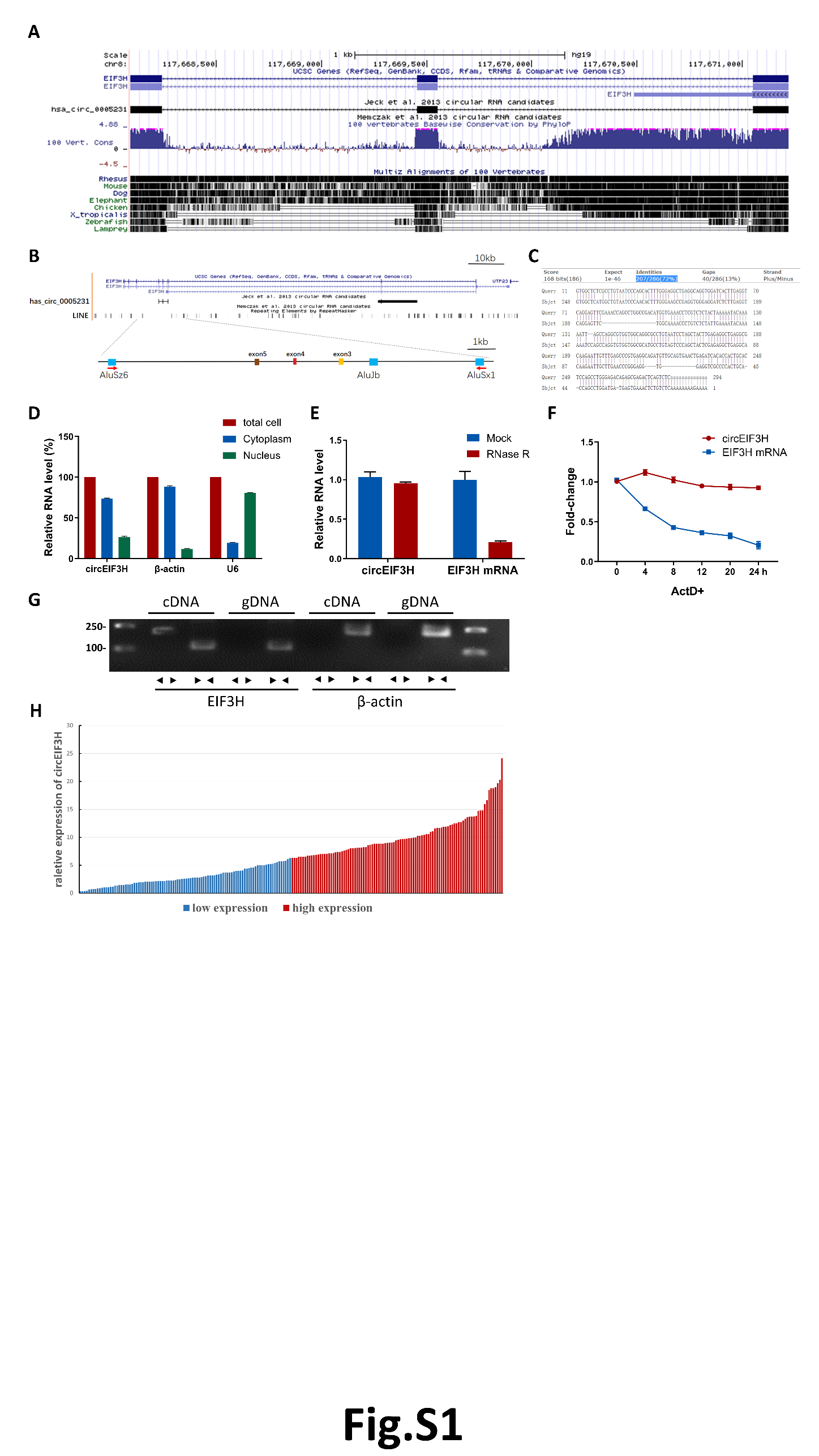


**Fig. S1 The genomic locus, characteristics and tissue expression of circEIF3H**

**A** Schematic diagram showing the conservation of circEIF3H. **B** Schematic diagram showing the genomic locus and the long flanking introns with ALU sequence of circEIF3H. **C** Complementary ALU sequence (AluSz6 and AluSx1) in the long flanking introns of EIF3H exon 3 to exon 5. **D** The expression level of circEIF3H in the subcellular fractions of MDA-MB-231 was detected by qRT-PCR. U6 and GAPDH were used as nuclear and cytoplasmic markers, respectively. **E** Total RNAs were digested with RNase R and qRT-PCR was used to detect circEIF3H and EIF3H mRNA after digestion. **F** The expression of circEIF3H and EIF3H mRNA in breast cancer cells treated with the transcription inhibitor Actinomycin D (2 μg/ml) at the indicated time points was detected by qRT-PCR. **G** Convergent or divergent primers were used to validate the existence of circEIF3H in TNBC cells. **H** According to the median expression level of circEIF3H, 198 breast cancer patients were divided into two groups. The blue bars represented the low expression group, and the red bars represented the high expression group. Columns are the average of three independent experiments.


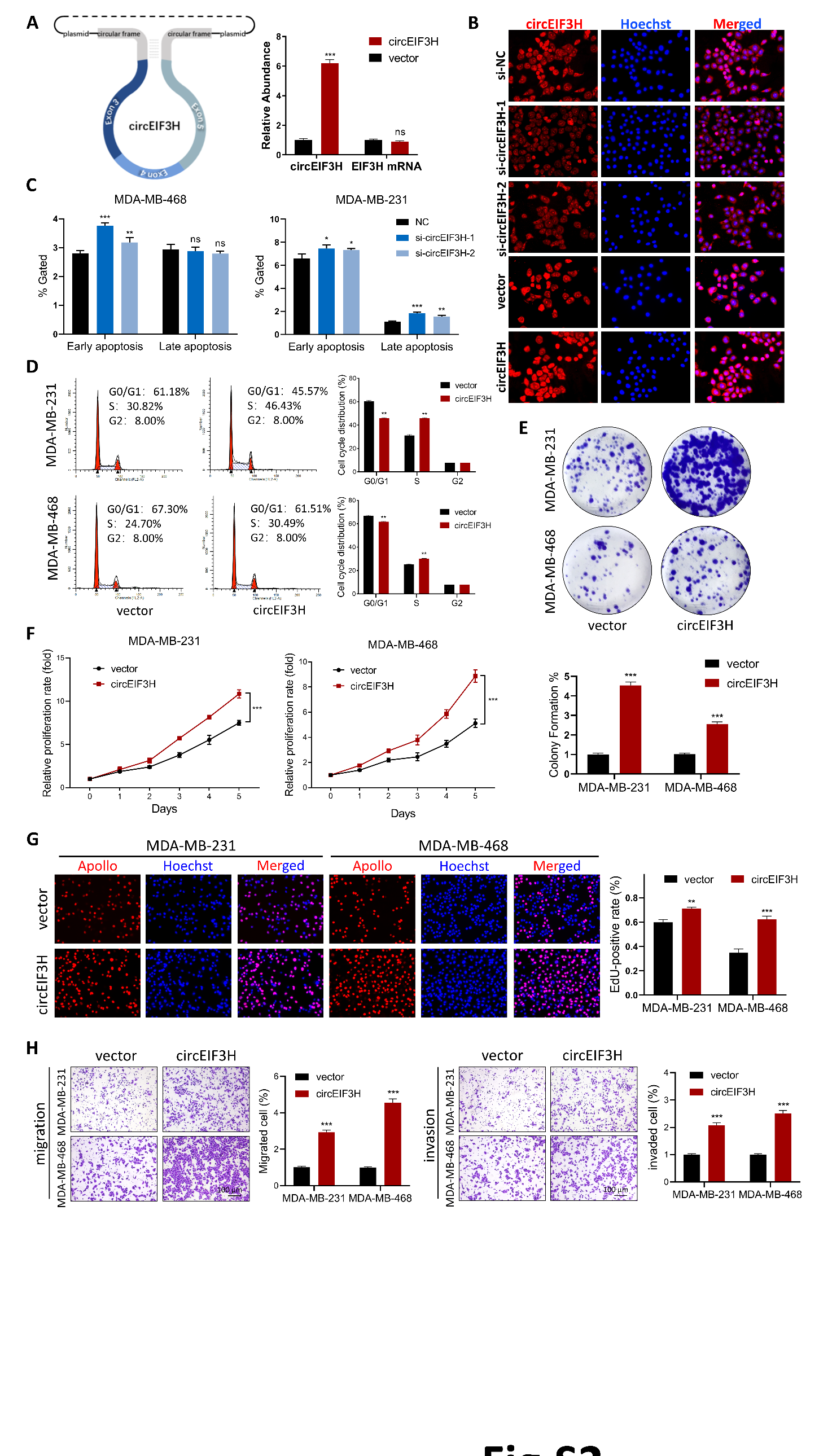


**Fig. S2 Overexpression of circEIF3H promoted the proliferation and metastasis of TNBC in vitro**

**A** Schematic illustration of circEIF3H overexpression plasmid and the overexpression efficacy measured by qRT-PCR. **B** The expression levels of circEIF3H after transfection with si-circEIF3H or circEIF3H overexpression vector were detected by FISH. **C** Apoptosis assay were conducted to detect the percentage of cell apoptosis after circEIF3H silencing. **D** Cell cycle distributions in circEIF3H overexpression cells were presented by flow cytometry. **E** Colony formation assays after circEIF3H overexpression **F-G** The effects of circEIF3H overexpression on cell proliferation examined by MTT assay (F) and EdU (G). **H** Transwell migration and invasion assays were used to evaluate the motility of MDA-MB-231 and MDA-MB-468 cells with circEIF3H overexpression. Columns are the average of three independent experiments. ***P* < 0.01, ****P* < 0.001.


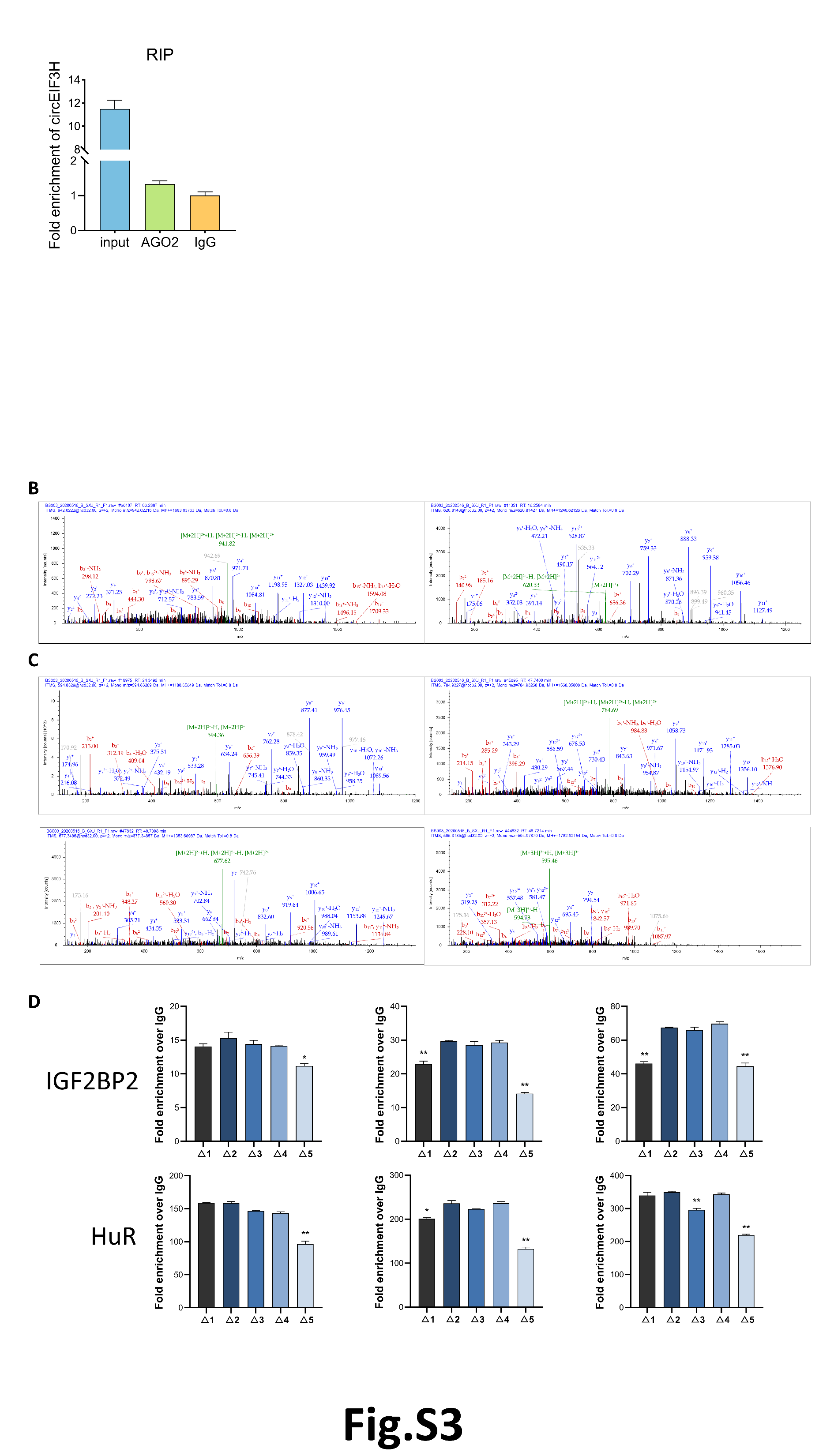


**Fig. S3 CircEIF3H interacts with IGF2BP2 and HuR as a scaffold**

RIP assay showed no significant enrichment of circEIF3H with AGO2.


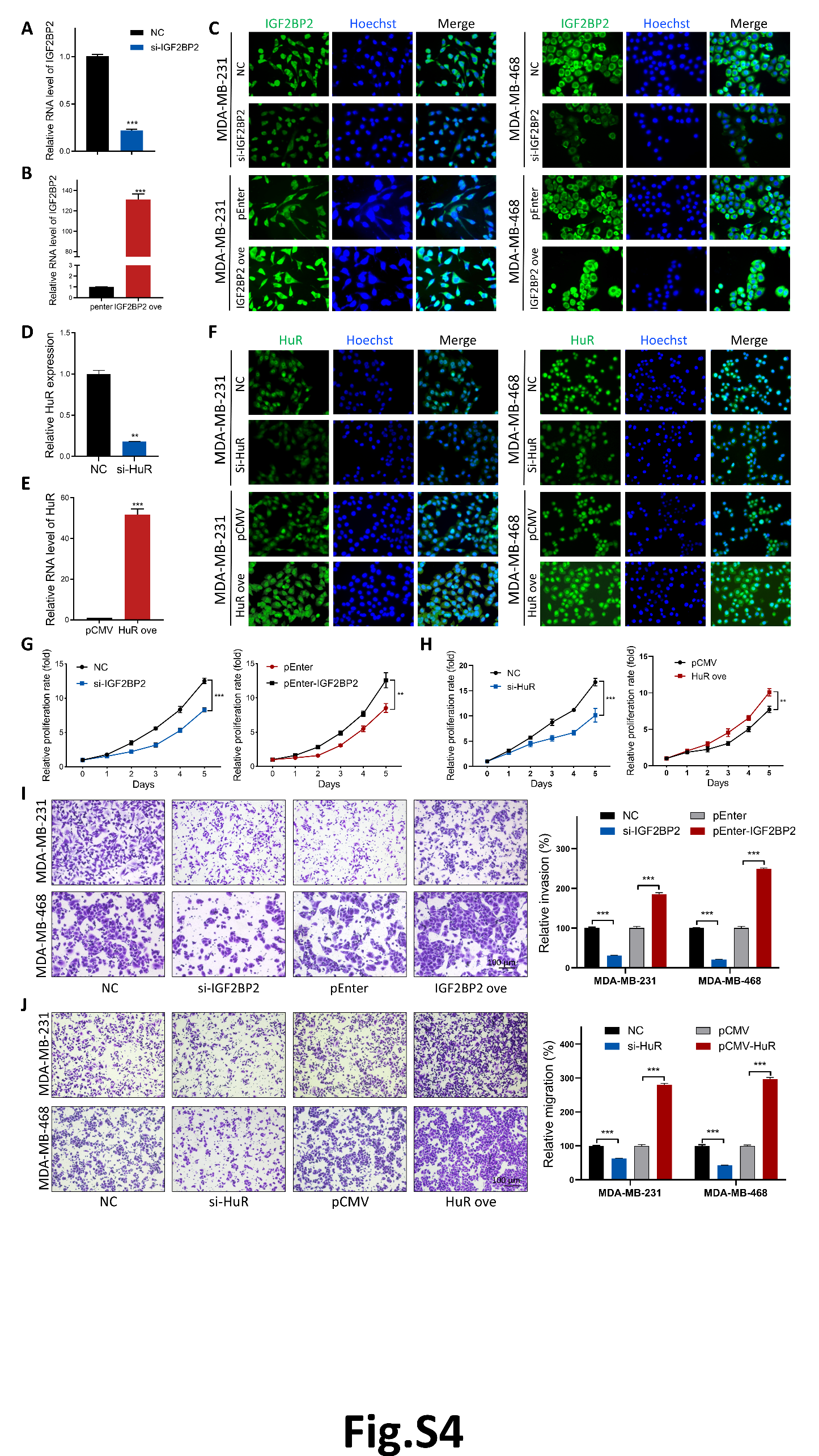


**Fig. S4 Both IGF2BP2 and HuR promoted TNBC progression in vitro**

**A-C** Transfection efficacy of si-IGF2BP2 and IGF2BP2 overexpression plasmid were detected by qRT-PCR (A-B) and immunofluorescence (C). **D-F** Transfection efficacy of si-HuR and HuR overexpression plasmid were detected by qRT-PCR (D-E) and immunofluorescence (F). **G** The effect of IGF2BP2 on TNBC proliferation was detected by MTT assay. **H** The effect of HuR on TNBC proliferation was detected by MTT assay. **I** The effect of IGF2BP2 on cell migration and invasion were detected by transwell system. **J** The effect of HuR on cell migration and invasion were detected by transwell system. Each column is the average of three independent experiments. ***P* < 0.01, ****P* < 0.001.


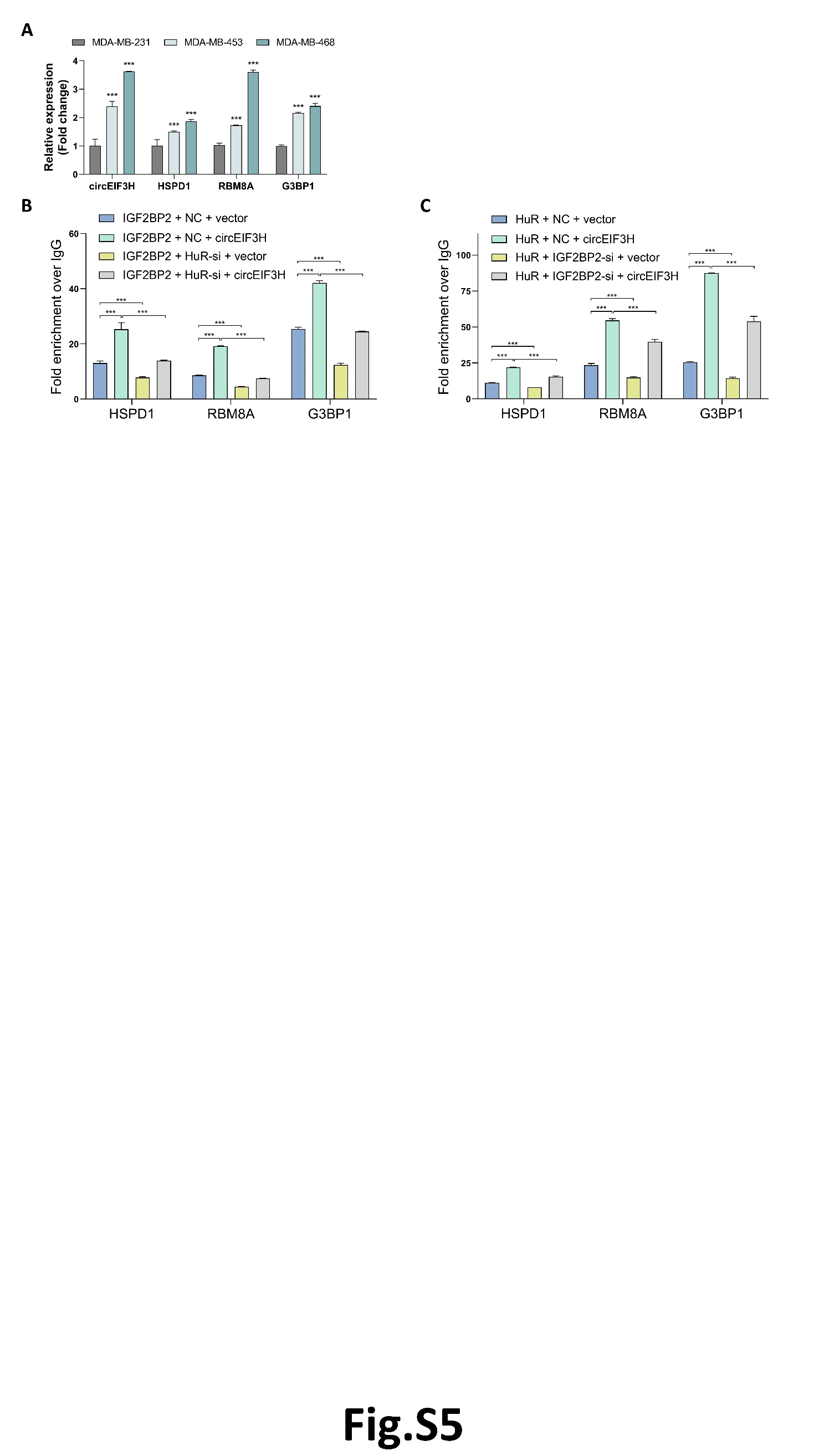


**Fig. S5 CircEIF3H-IGF2BP2/HuR complex stabilized HSPD1/RBM8A/G3BP1 mRNA**

**A** Expression levels of circEIF3H, HSPD1, RBM8A and G3BP1 in 3 different TNBC cell lines. **B** RIP assays showed the decreased enrichment of HSPD1/RBM8A/G3BP1 with Flag-tagged IGF2BP2 after si-HuR transfection and increased enrichment after circEIF3H overexpression. **C** RIP assays showed the decreased enrichment of HSPD1/RBM8A/G3BP1 with HA-tagged HuR after si-IGF2BP2 transfection and increased enrichment after circEIF3H overexpression. ****P* < 0.001.


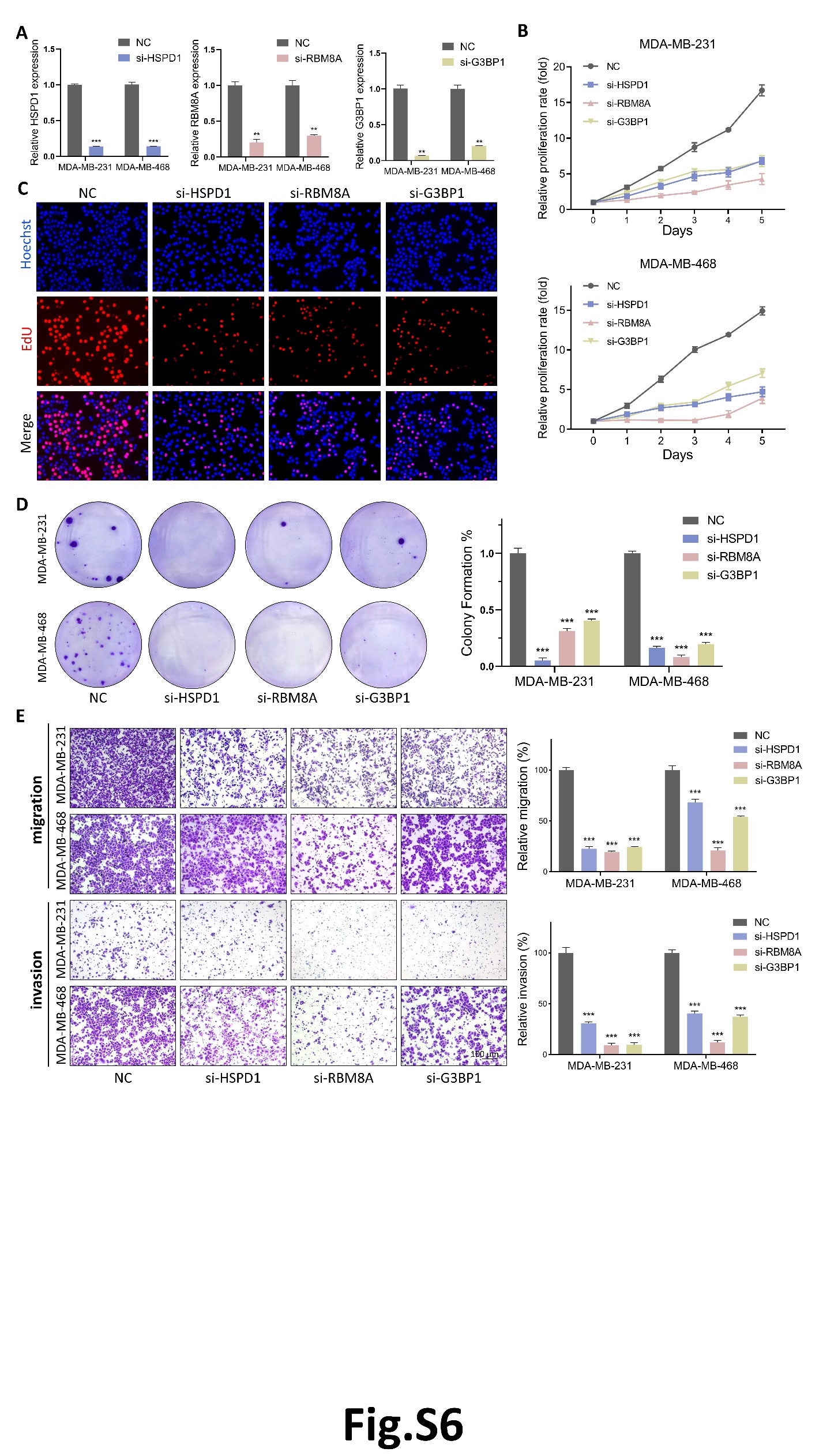


**Fig. S6 HSPD1, RBM8A and G3BP1 were essential for TNBC proliferation and metastasis**

**A** The efficacy of HSPD1/RBM8A/G3BP1 siRNA verified by qRT-PCR. **B-D** The effects of HSPD1/RBM8A/G3BP1 knockdown on the proliferation of TNBC cells were examined by MTT assay (B), EdU assay (C) and colony formation assays (D). **(E)** Transwell assays were used to measure the migration and invasion of breast cancer cells with HSPD1/RBM8A/G3BP1 knockdown. Each column is the average of three independent experiments. ***P* < 0.01, ****P* < 0.001.


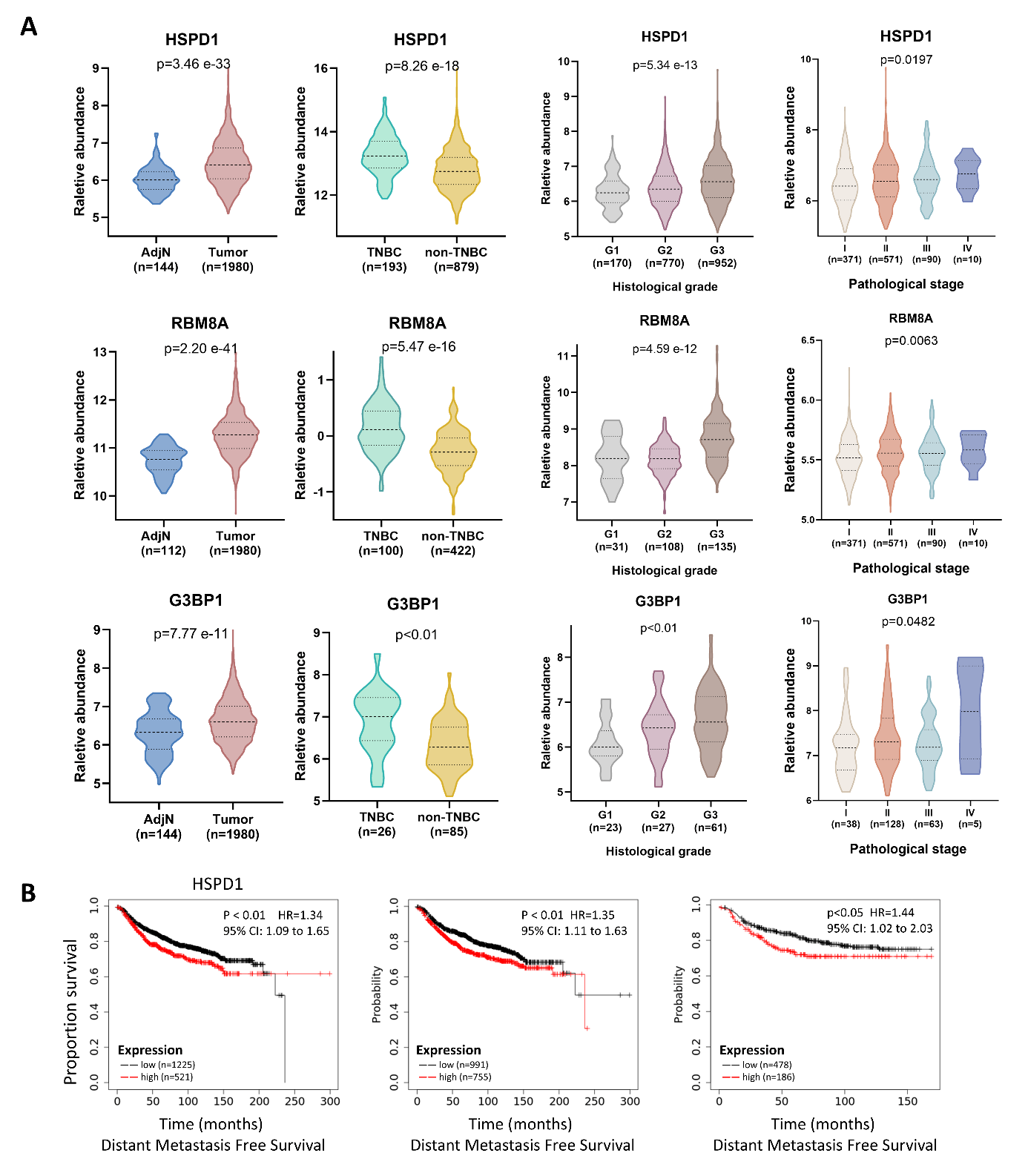


**Fig. S7 The association between HSPD1/RBM8A/G3BP1 expression and clinicopathological characteristics or prognosis in breast cancer patients**

**A** The association between HSPD1/RBM8A/G3BP1 expression and clinicopathological characteristics of breast cancer patients. **B** The association between HSPD1/RBM8A/G3BP1 expression and prognosis in breast cancer patients.


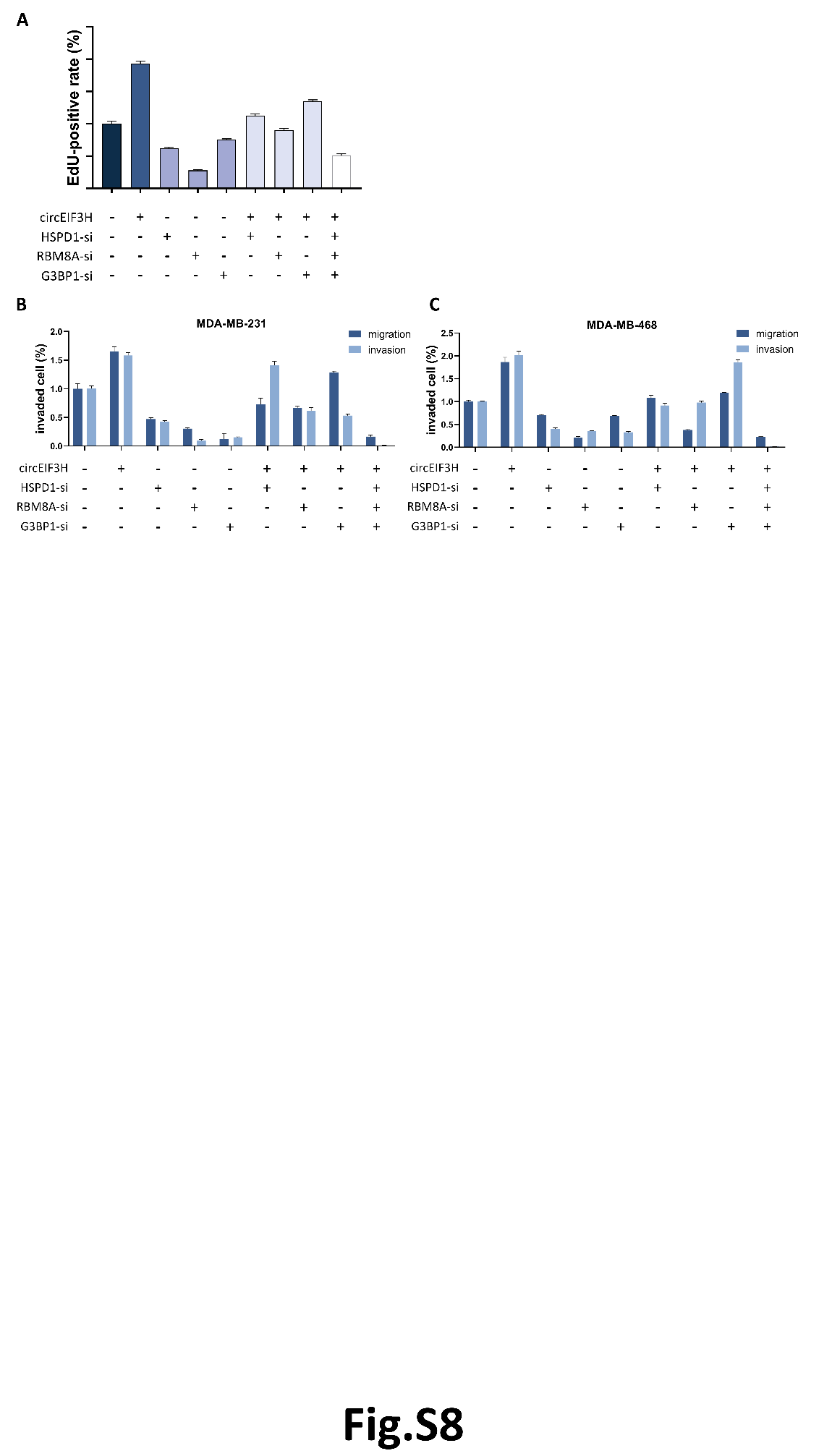


**Fig. 8. HSPD1/RBM8A/G3BP1 silence rescues the cancer promoting effect of circEIF3H**

**A-C** After cotransfection with circEIF3H overexpression plasmid and HSPD1/RBM8A/G3BP1 siRNAs respectively, cell proliferation was detected via EdU (A), metastatic capacity was detected via transwell migration (B) and invasion assay (C). Each column is the average of three independent experiments.
