# Supplementary material for: CircEIF3H-IGF2BP2-HuR scaffold complex promotes TNBC progression via stabilizing HSPD1/RBM8A/G3BP1 mRNA
Source: Cell Death Discov. 2022 May 14;8:261. doi: 10.1038/s41420-022-01055-9 (PMC9107465; doi:10.1038/s41420-022-01055-9)
Supplement: Supplementary file 2 — Supplementary table [file 41420_2022_1055_MOESM2_ESM.docx]

**Supplementary Tables**

**Table S1. Primer sequences used in this manuscript.**

| **Primer name** | **Forward primer (5’-3’)** | **Reverse primer (5’-3’)** |
| --- | --- | --- |
| *EIF3H* | GATTCAGCCGTGAAGCAAGTGC | CCAGACCCAAAAGCACTCCTTG |
| circEIF3H convergent | ATGGAAATGATGCGGAGCCT | TAAACTGAGAGTCCAGGAGTG |
| circEIF3H divergent | GTCCTAATGTGGGAACTT | ATGCTGGTAACTAAACTGA |
| IGF2BP2 | GGCTCCCTGATCTGGTTAAGGA | CCACTTCCATTCTGATGACCAGC |
| HuR | TGTTCTCTCGGTTTGGGCGGAT | TCTTCTGCCTCCGACCGTTTGT |
| HSPD1 | TGCCAATGCTCACCGTAAGCCT | AGCCTTGACTGCCACAACCTGA |
| RBM8A | GCGTGAGGATTATGACAGCGTG | TTCGGTGGCTTCCTCATGGACT |
| G3BP1 | AGCCTGTTCAGAAAGTCCTTAGC | CGAAGGCGATTATCTCGTCGGT |
| IGF2 | TGGCATCGTTGAGGAGTGCTGT | ACGGGGTATCTGGGGAAGTTGT |
| P21 | AGGTGGACCTGGAGACTCTCAG | TCCTCTTGGAGAAGATCAGCCG |
| β-actin | CATGTACGTTGCTATCCAGGC | CTCCTTAATGTCACGCACGAT |
| 18s rRNA | CGGACAGGATTGACAGATTGATAGC | TGCCAGAGTCTCGTTCGTTATCG |

**Table S2. siRNA sequences used in this manuscript.**

| **Name** | **Sequence (5'-3')** |
| --- | --- |
| si-circEIF3H-1 | CAGCAGUCCAAUAUCAGAU |
| si-circEIF3H-2 | CAGCCUUGCCAGCAGUCCA |
| si-IGF2BP2 | GCCGUUGUCAACGUCACAU |
| si-HuR | GGUUUGGGCGGAUCAUCAA |
| si-HSPD1 | GACGAUGCCAUGCUCUUAA |
| si-RBM8A | GGCUGGAUUCUCUUUGUAA |
| si-G3BP1 | GCGAGAACAACGAAUAAAU |
| negative control | UUCUCCGAACGUGUCACGU |
