# Supplementary material for: CircEIF3H-IGF2BP2-HuR scaffold complex promotes TNBC progression via stabilizing HSPD1/RBM8A/G3BP1 mRNA
Source: Cell Death Discov. 2022 May 14;8:261. doi: 10.1038/s41420-022-01055-9 (PMC9107465; doi:10.1038/s41420-022-01055-9)

Figure 3C

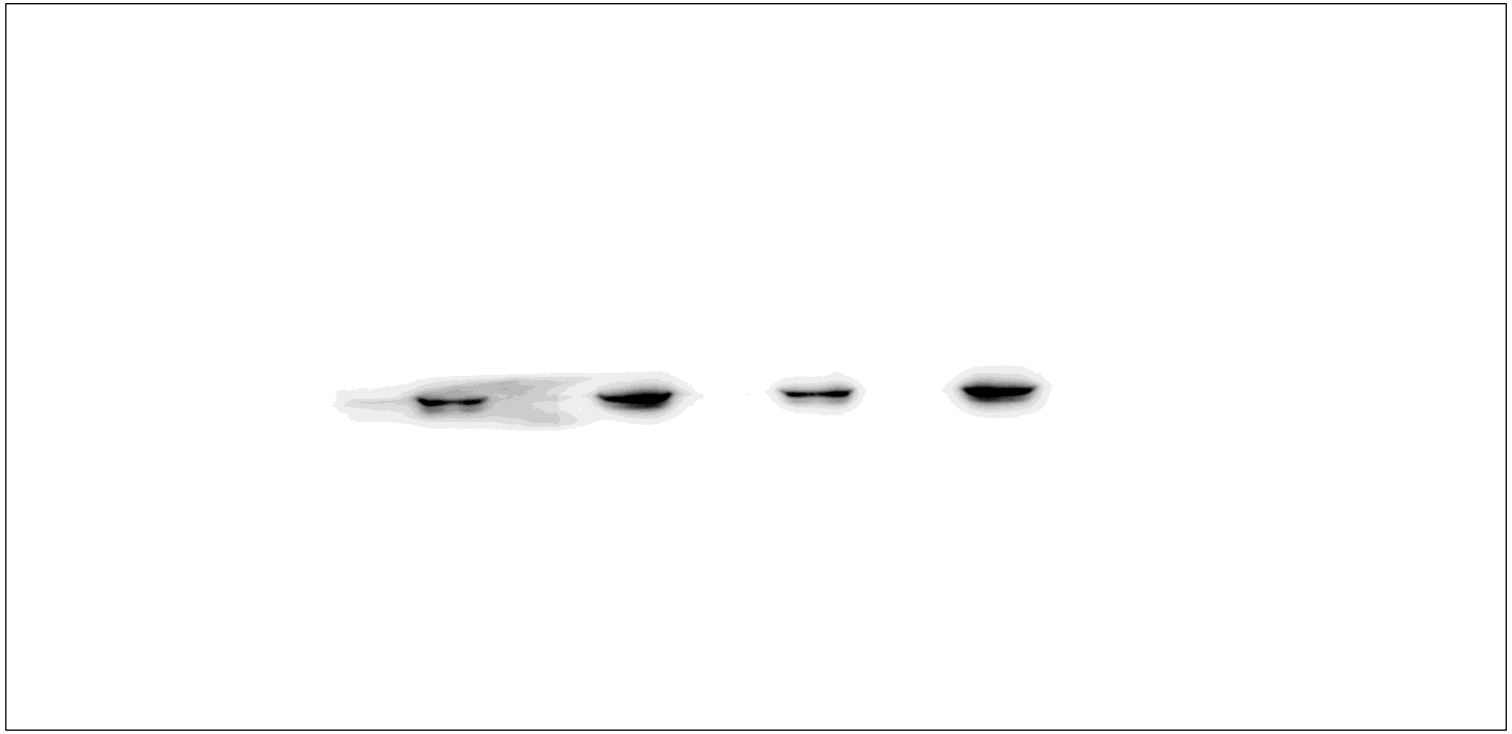

Figure 3C

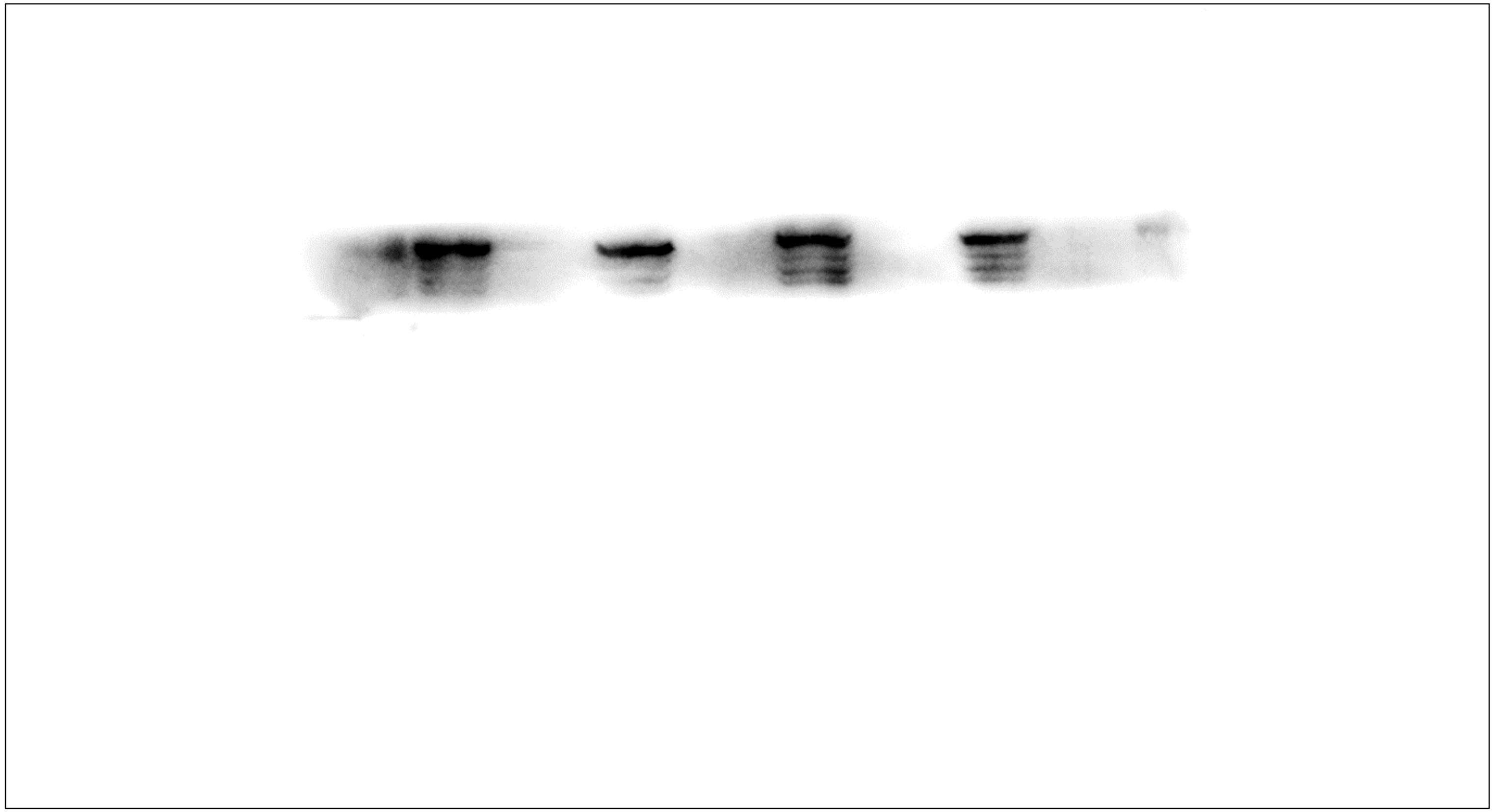

Figure 3D

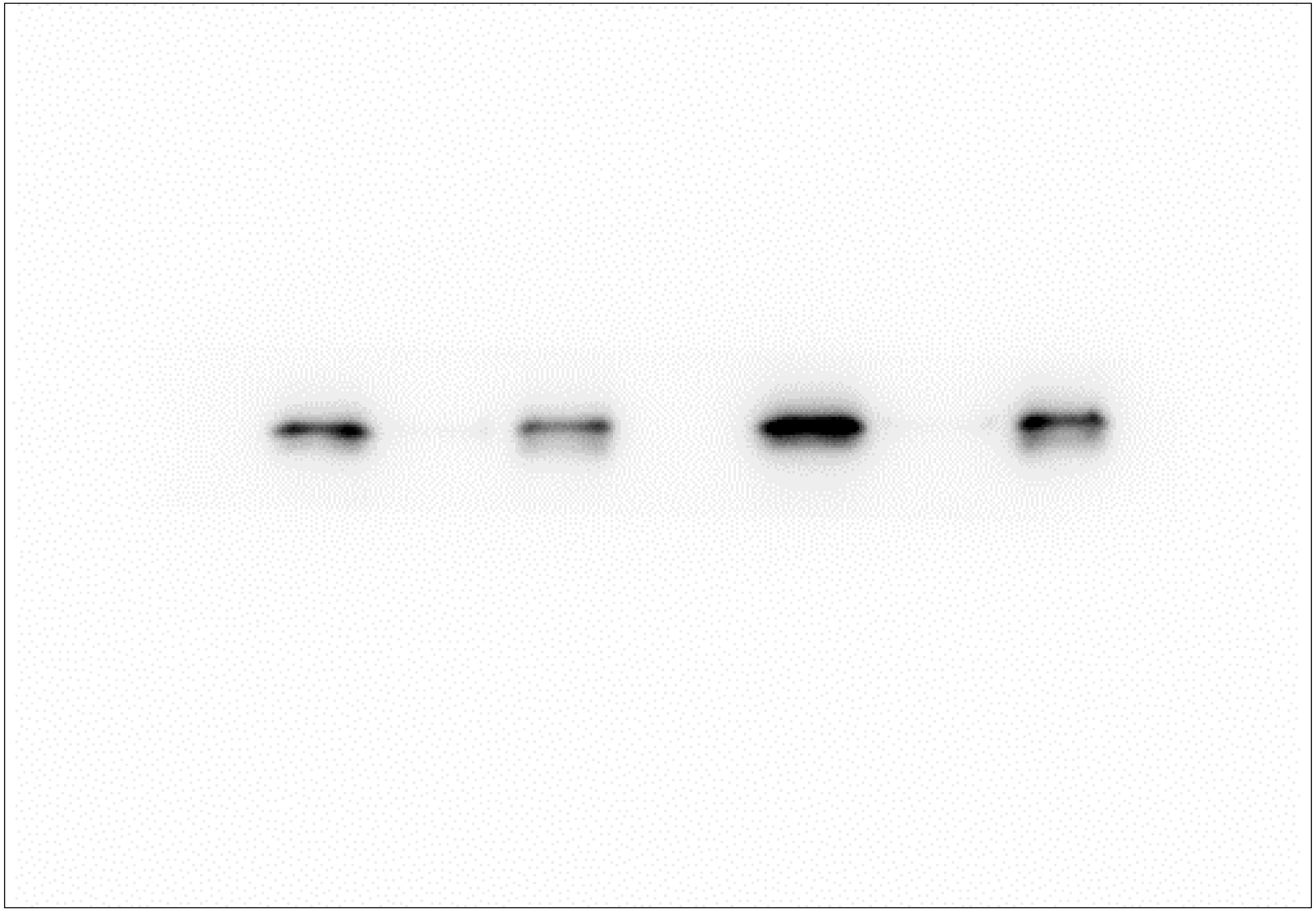

Figure 3D

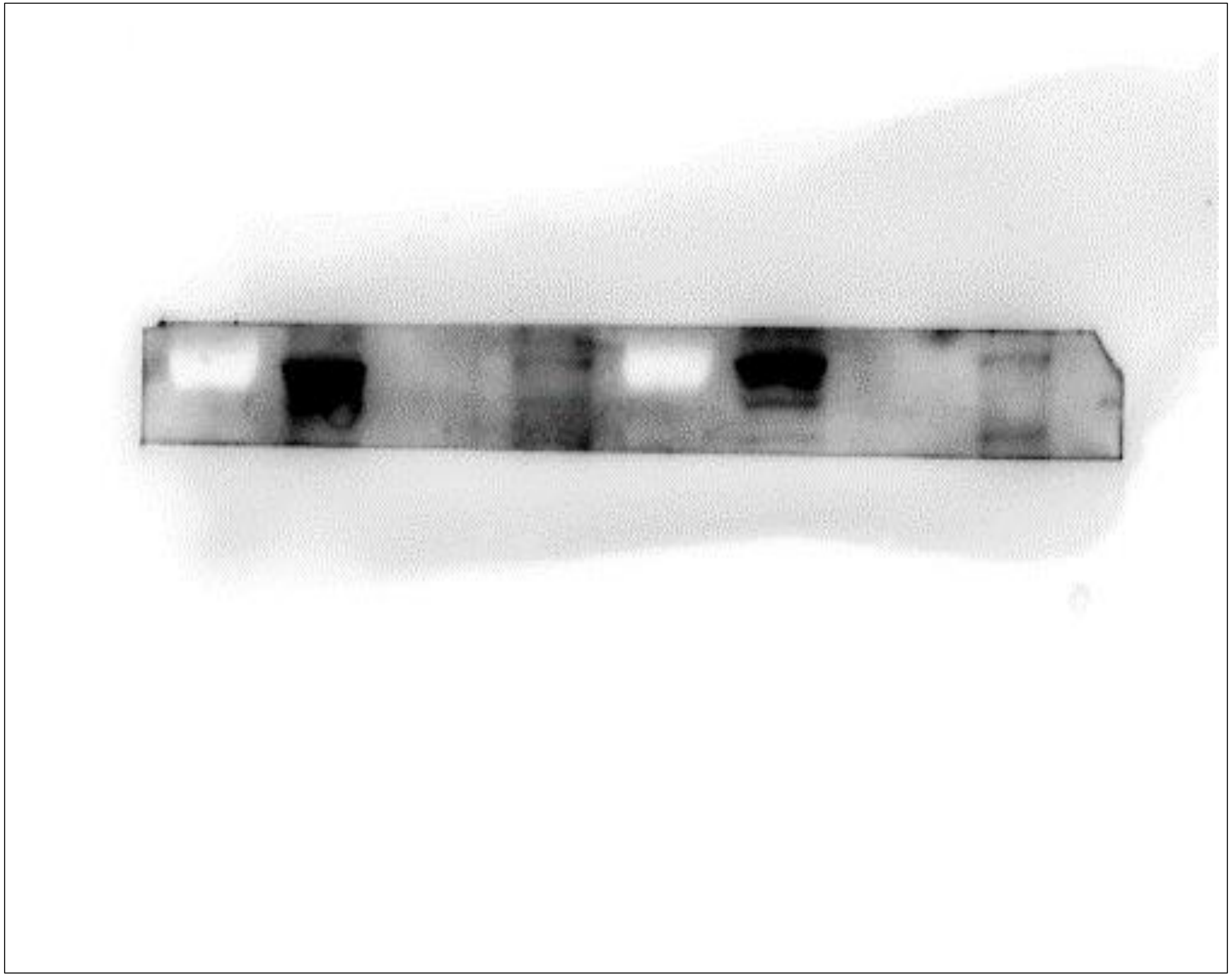

Figure 3G

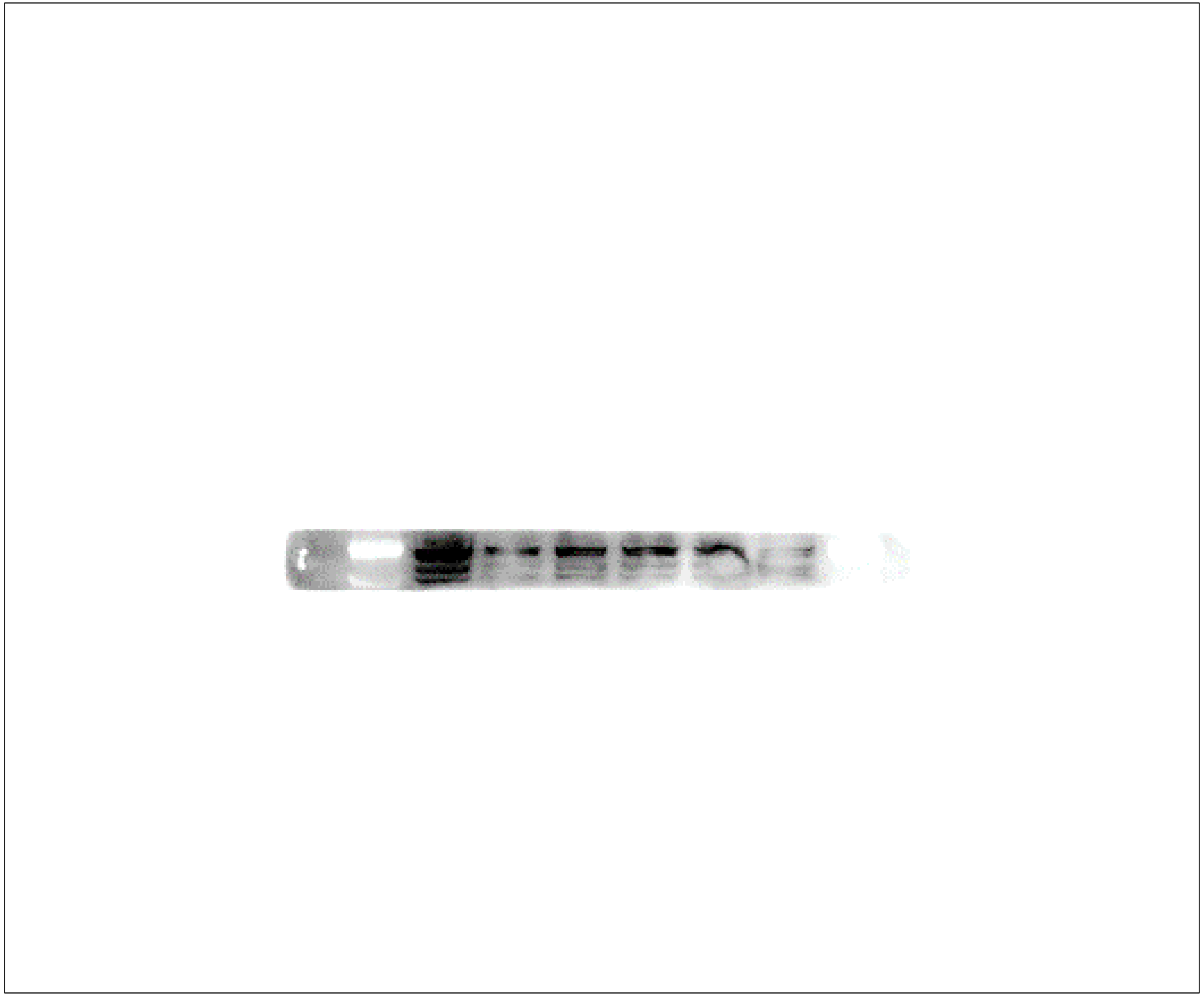

Figure 3G

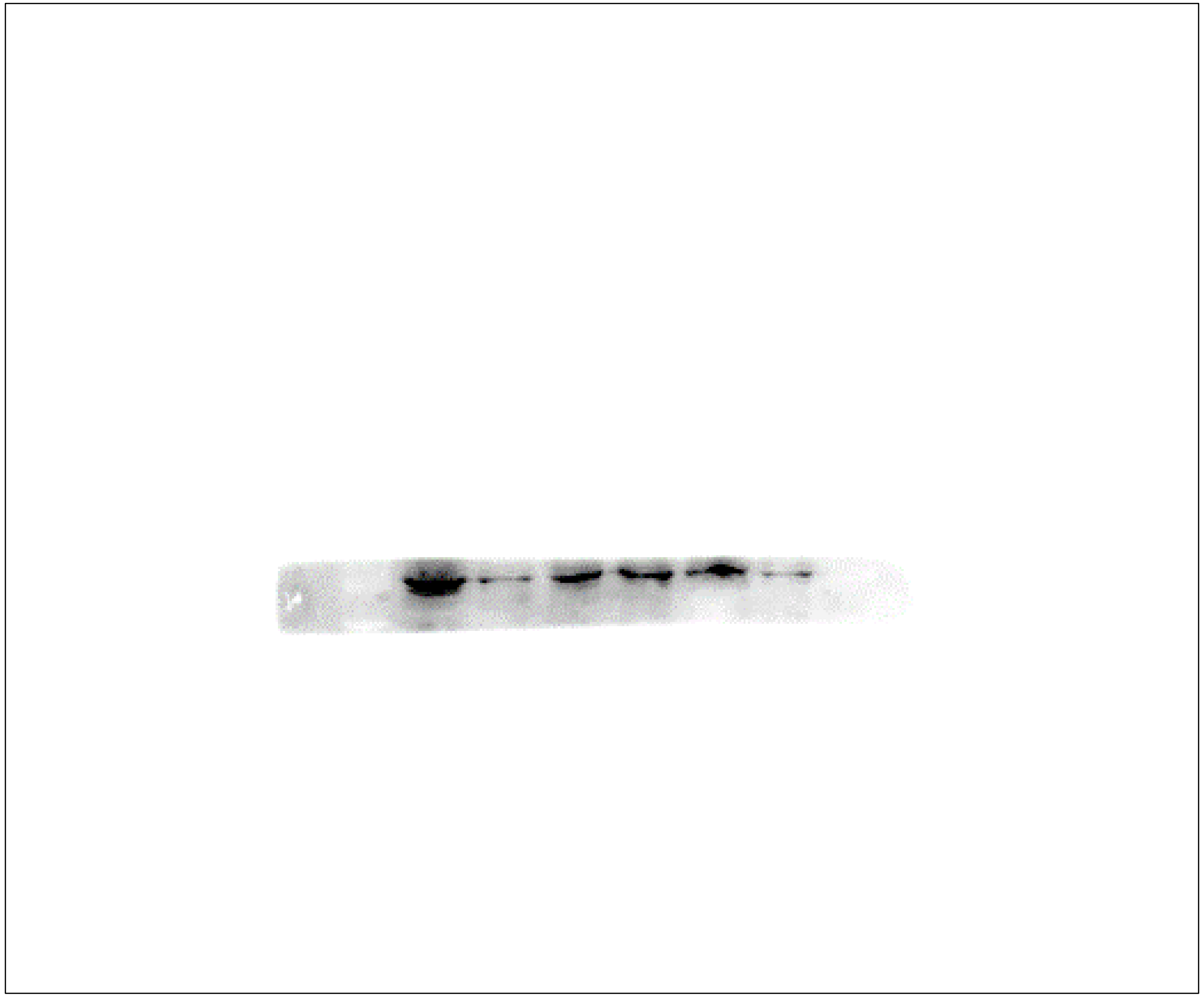

Figure 3G

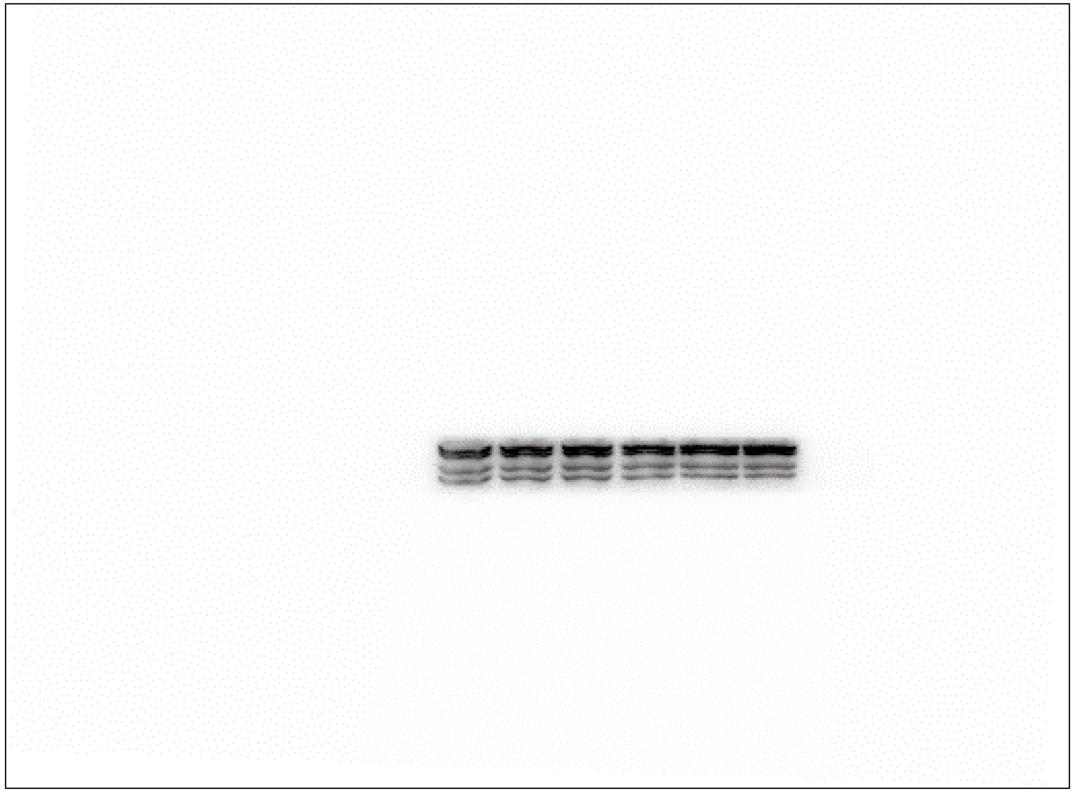

Figure 3G

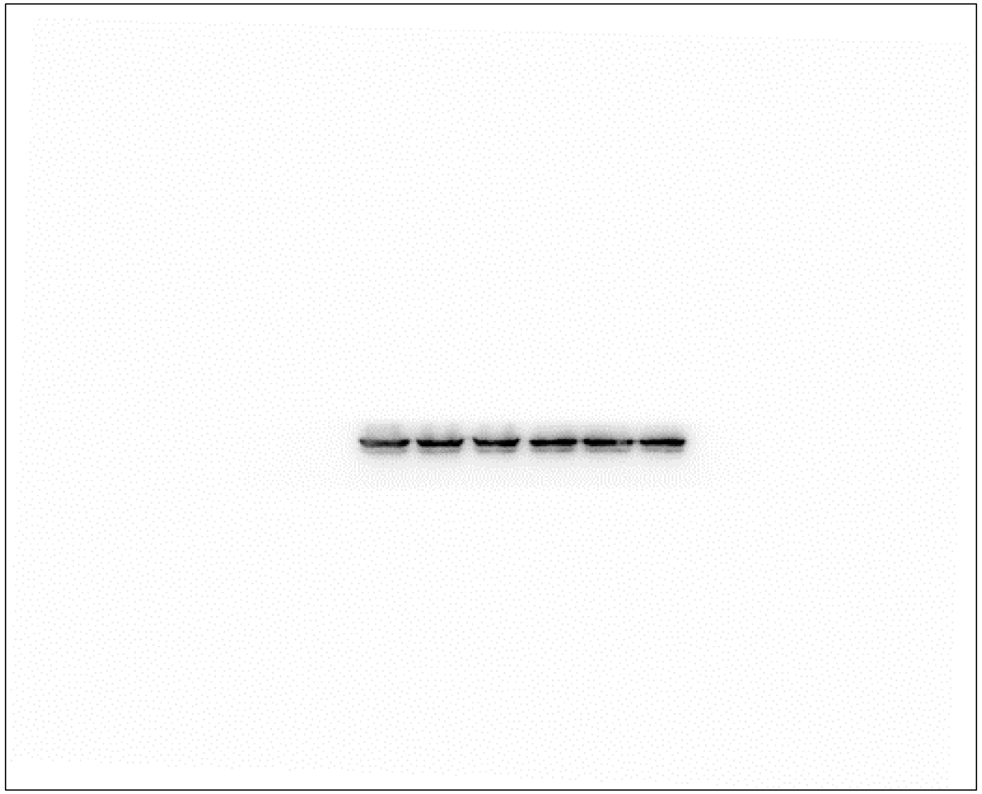

Figure 3G

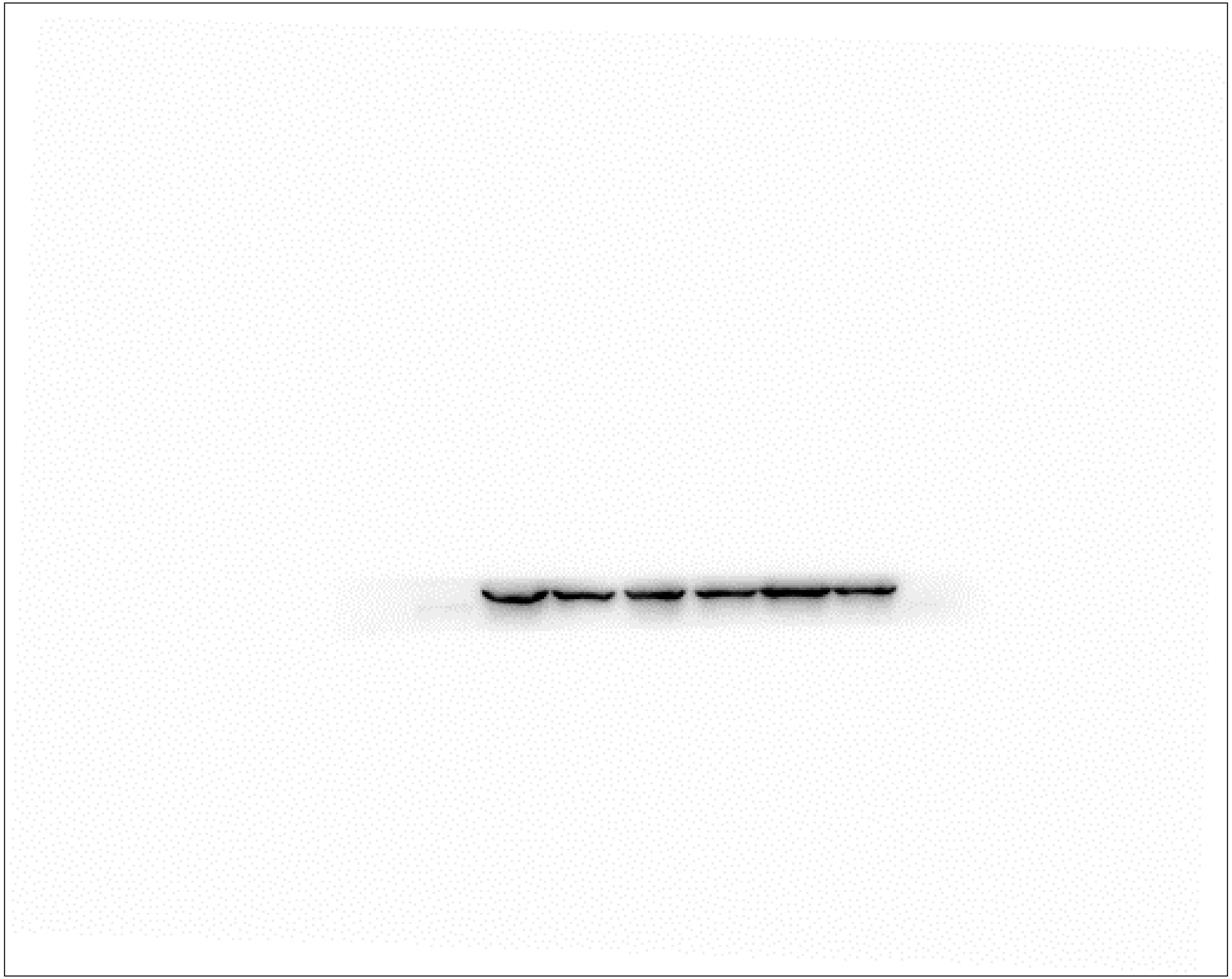

Figure 4C

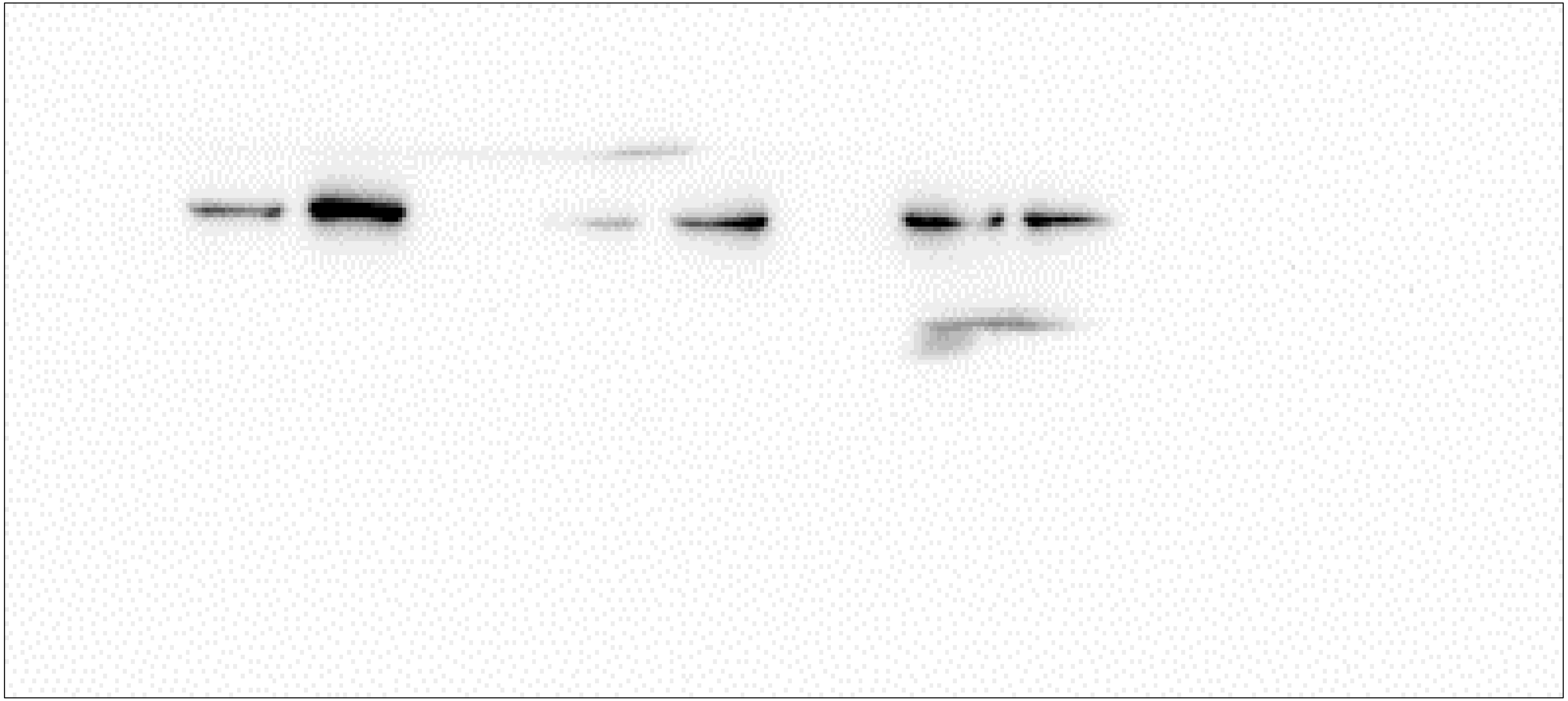

Figure 4C

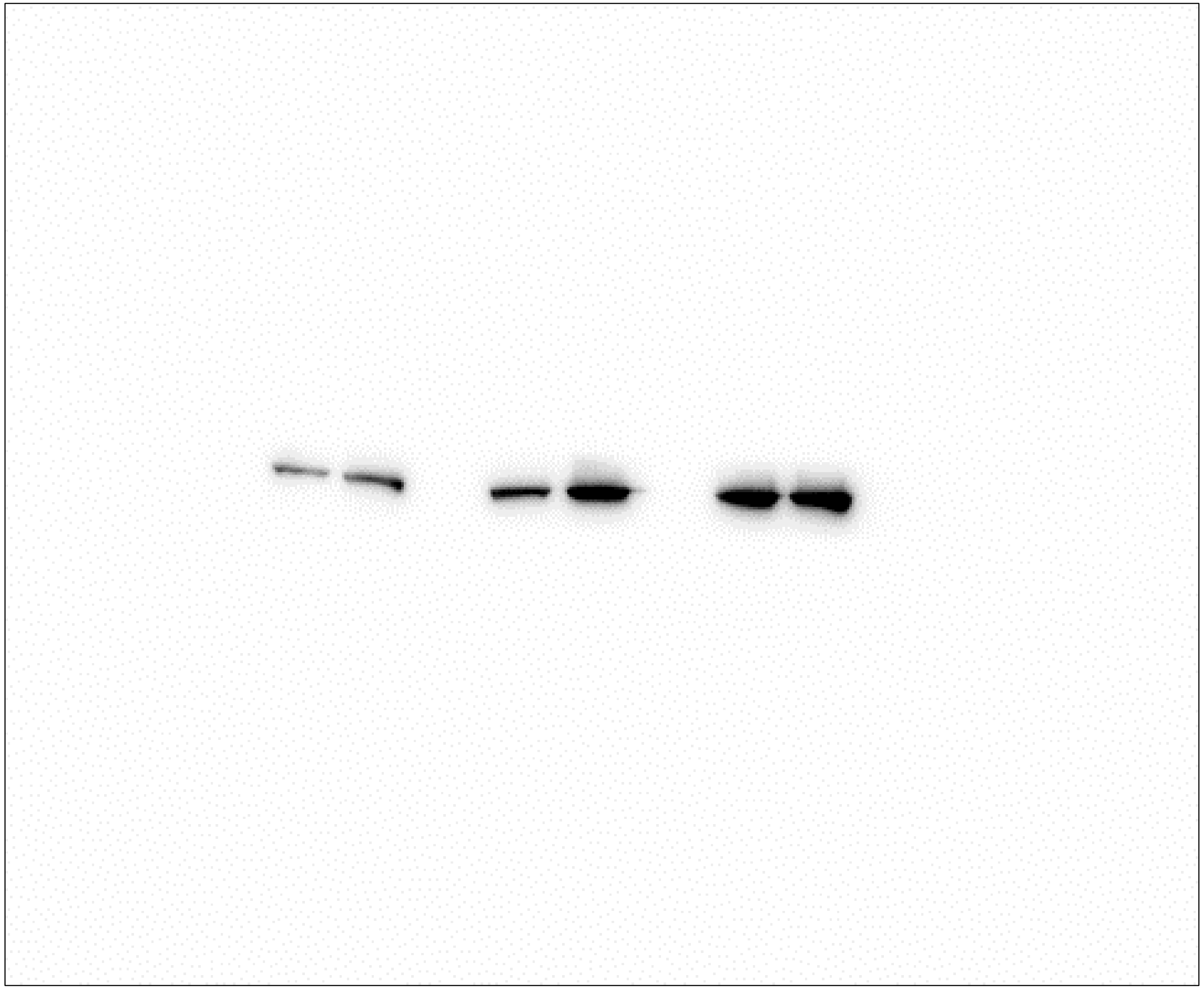

Figure 4C

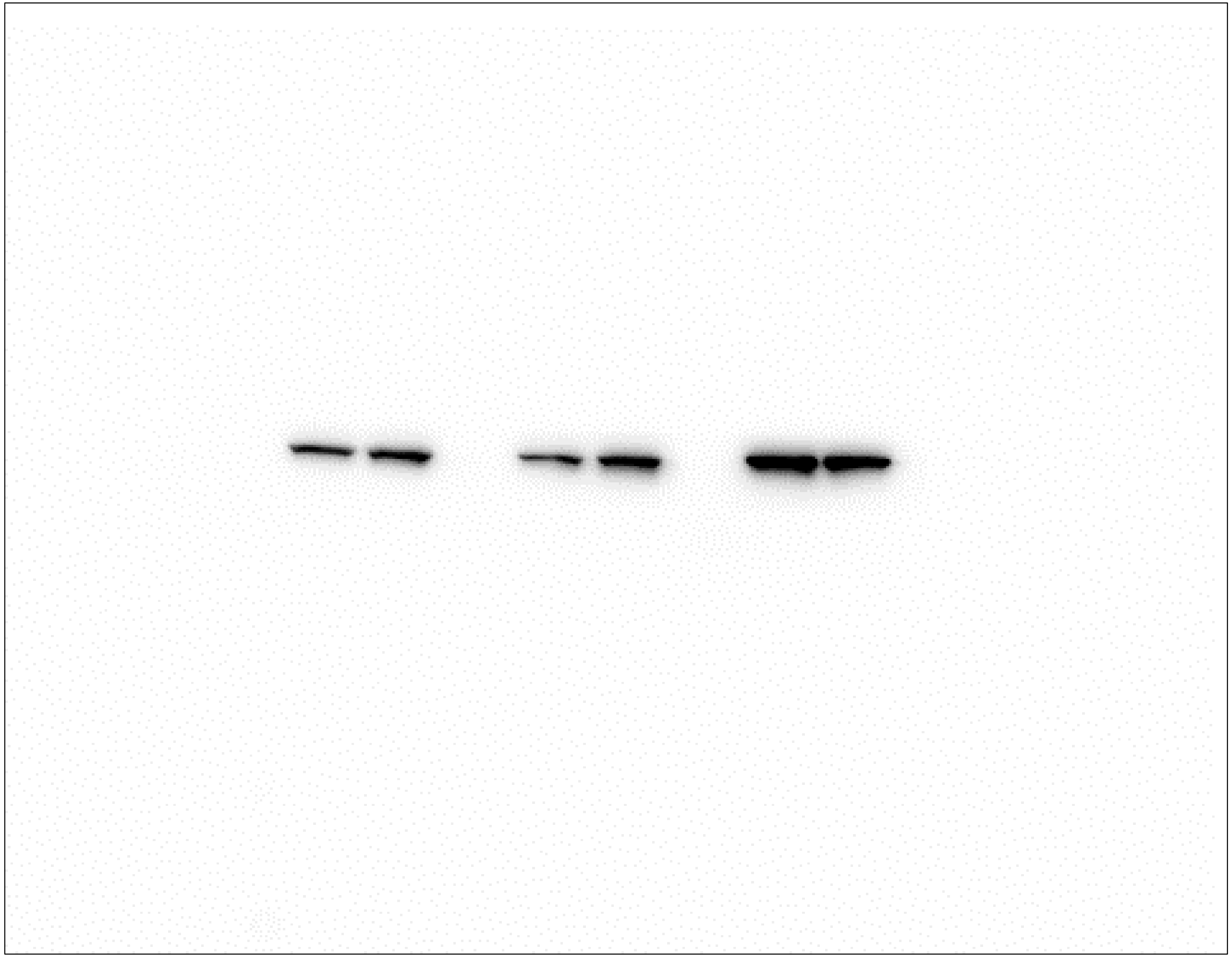

Figure 4C

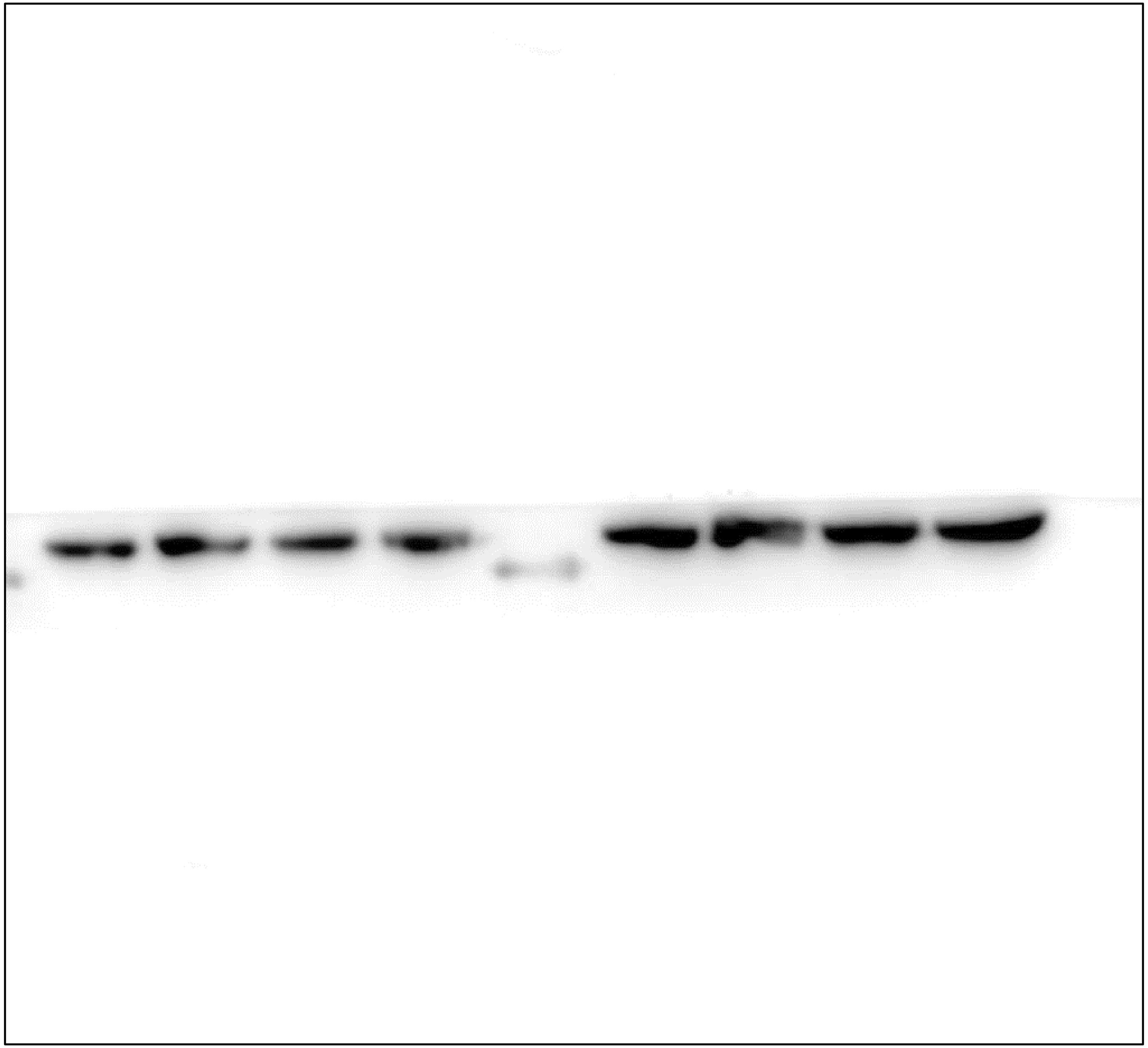

Figure 4C

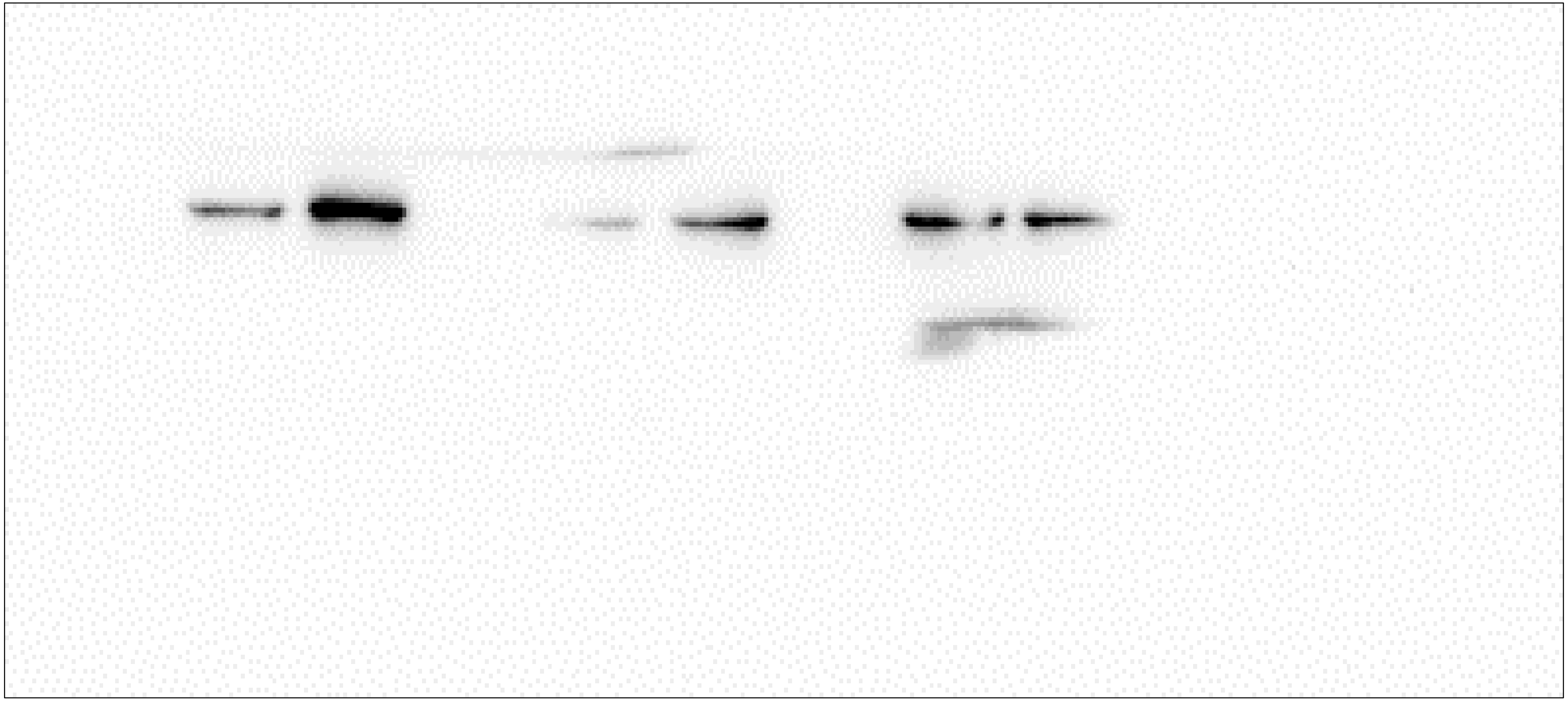

Figure 4C

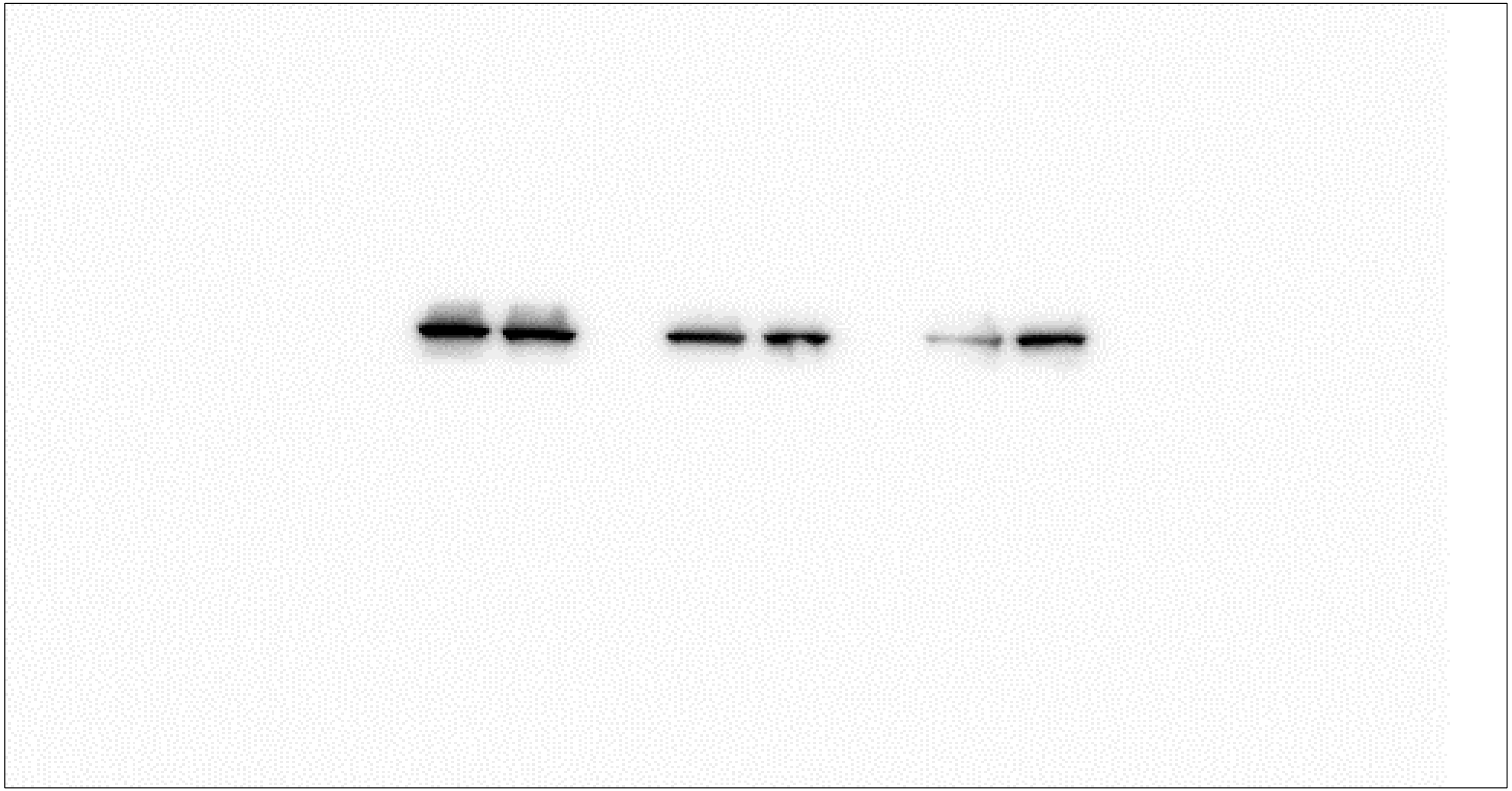

Figure 4C

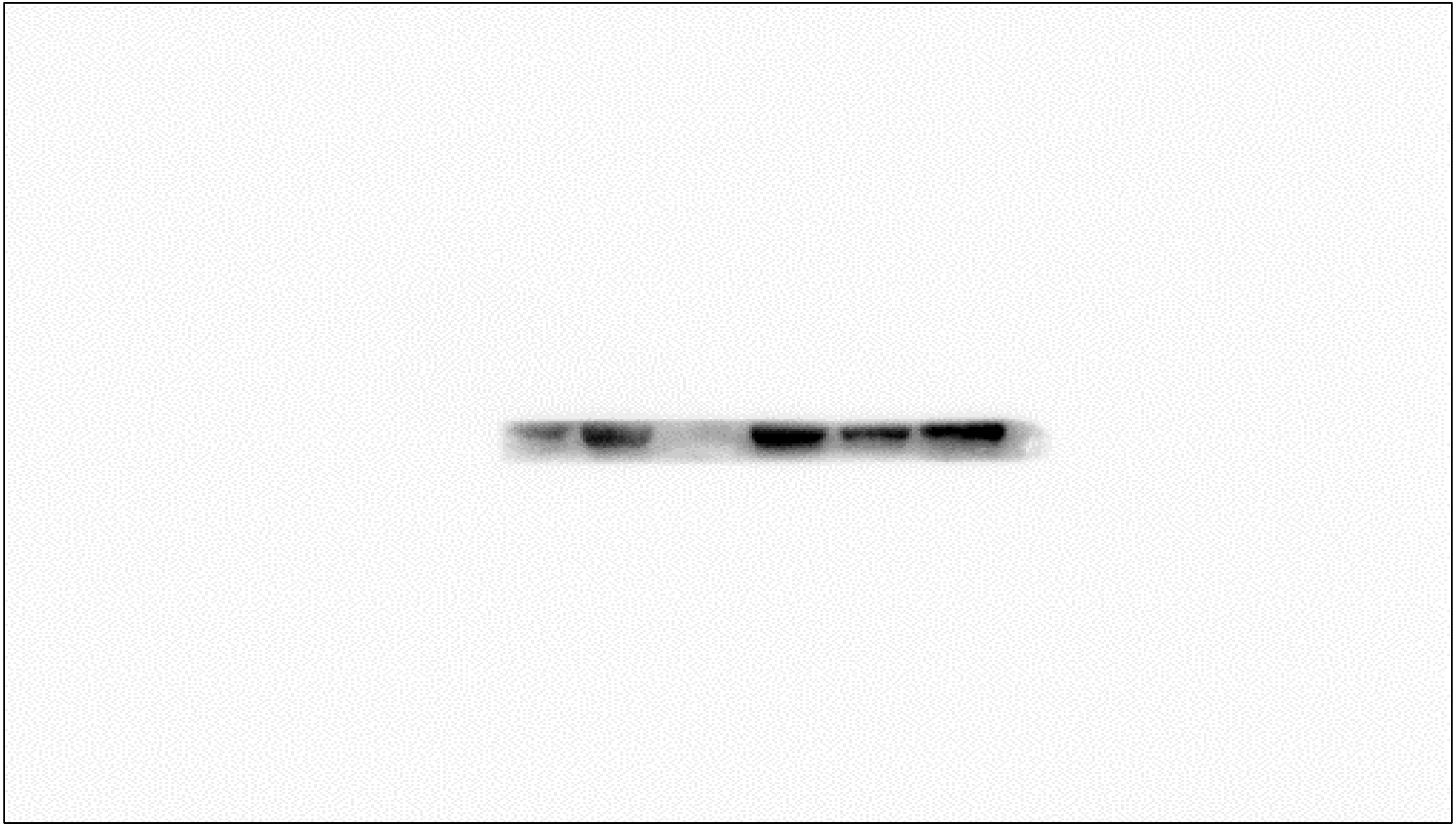

Figure 4C

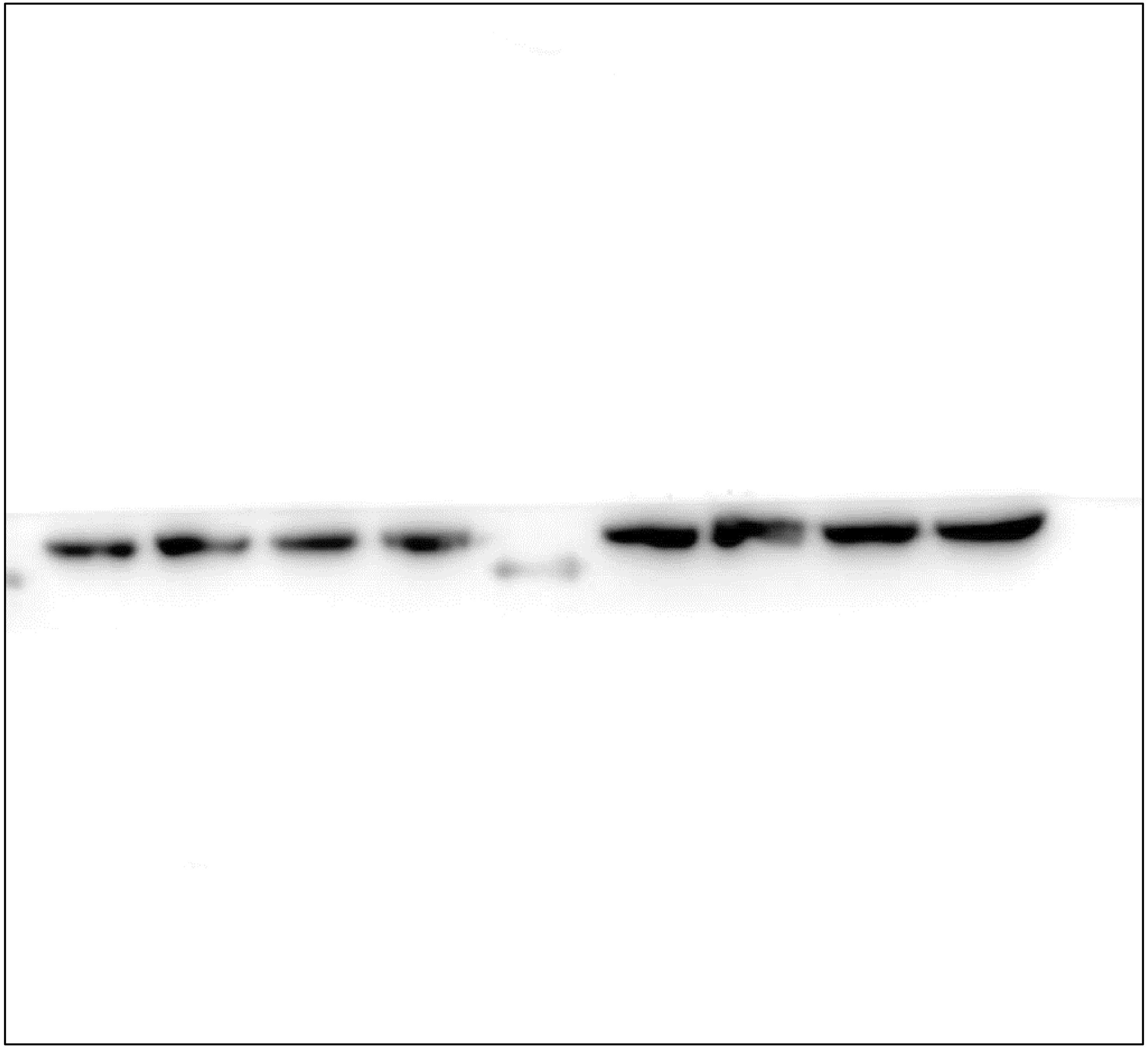

Figure 4D

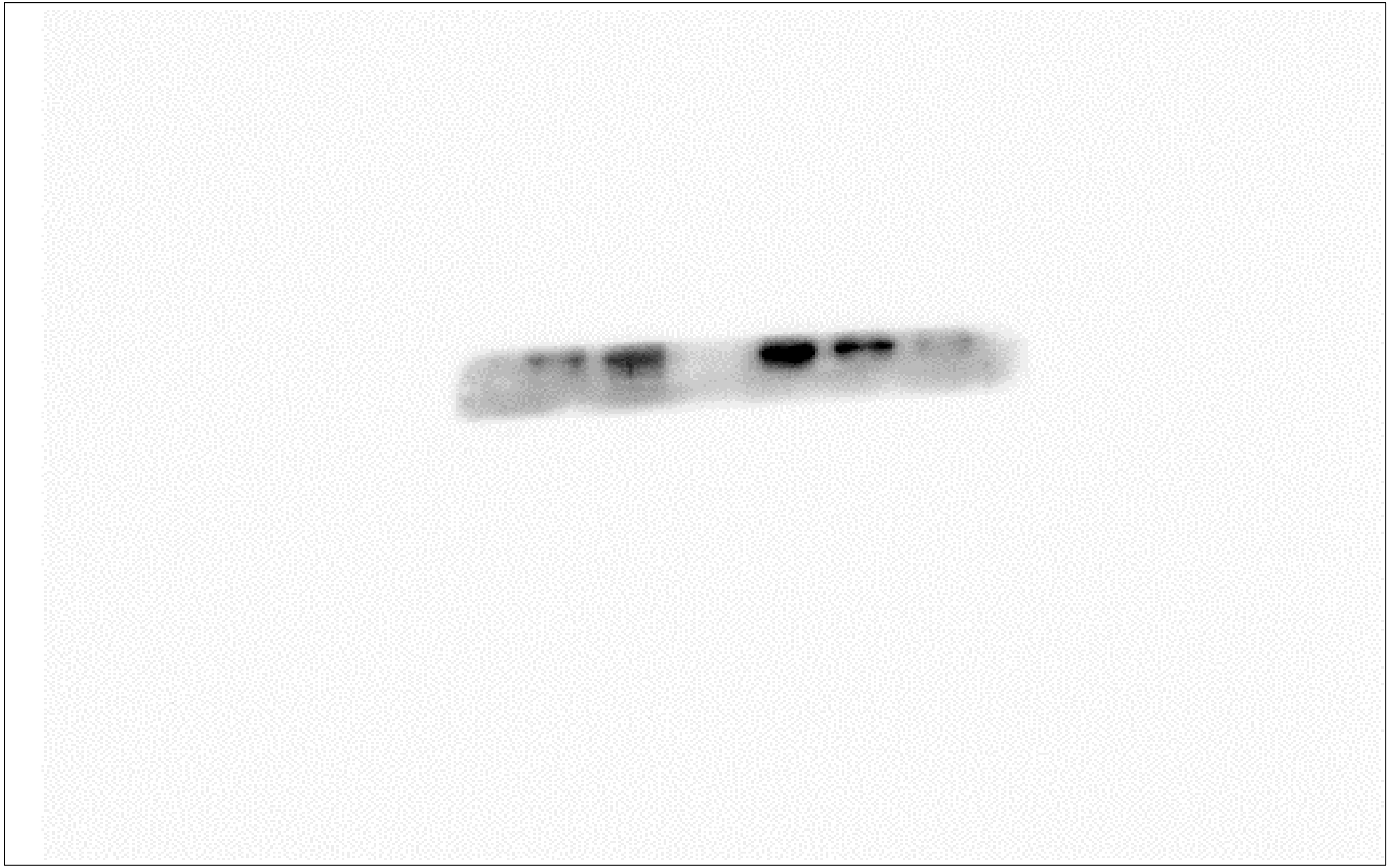

Figure 4D

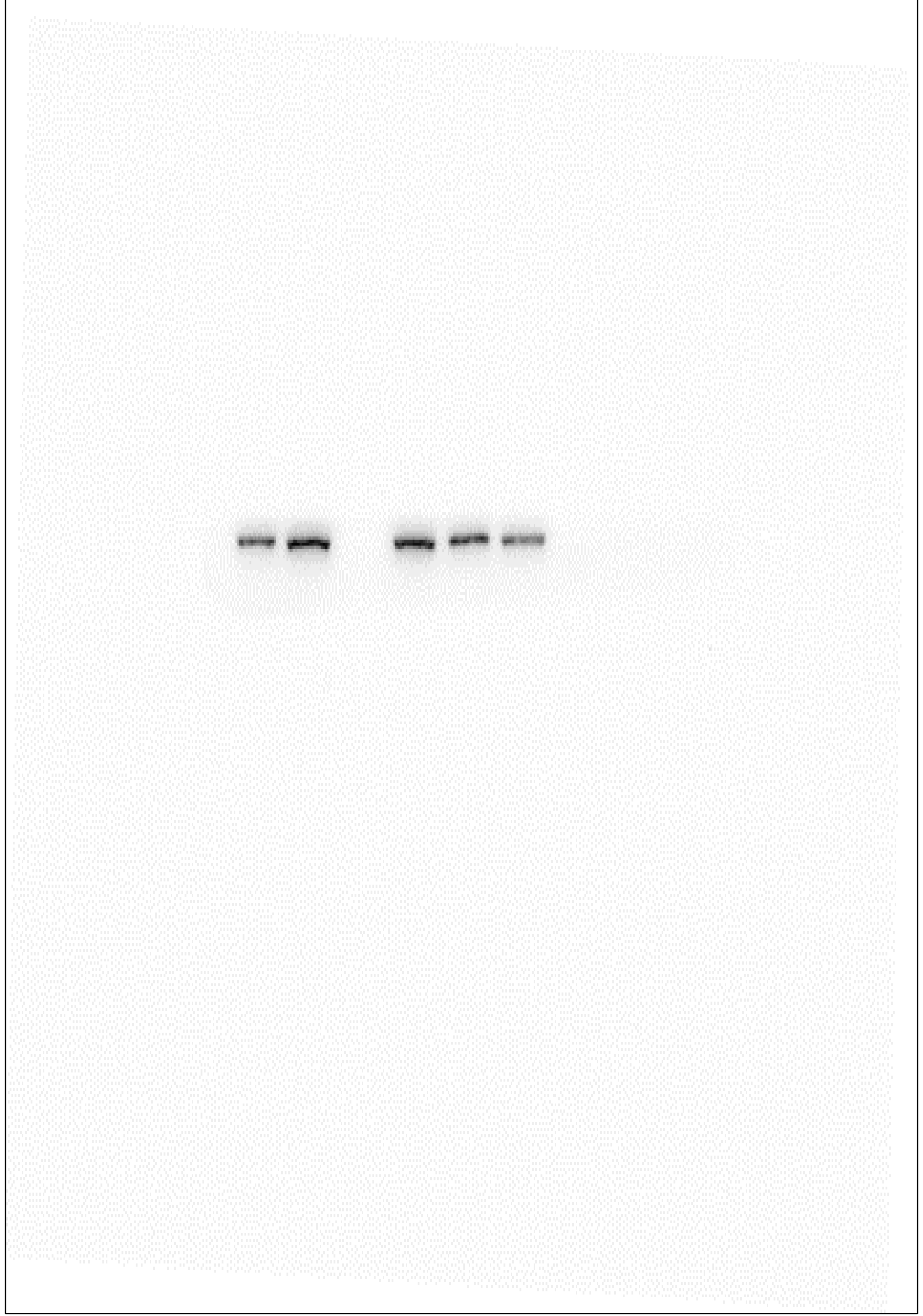

Figure 4D

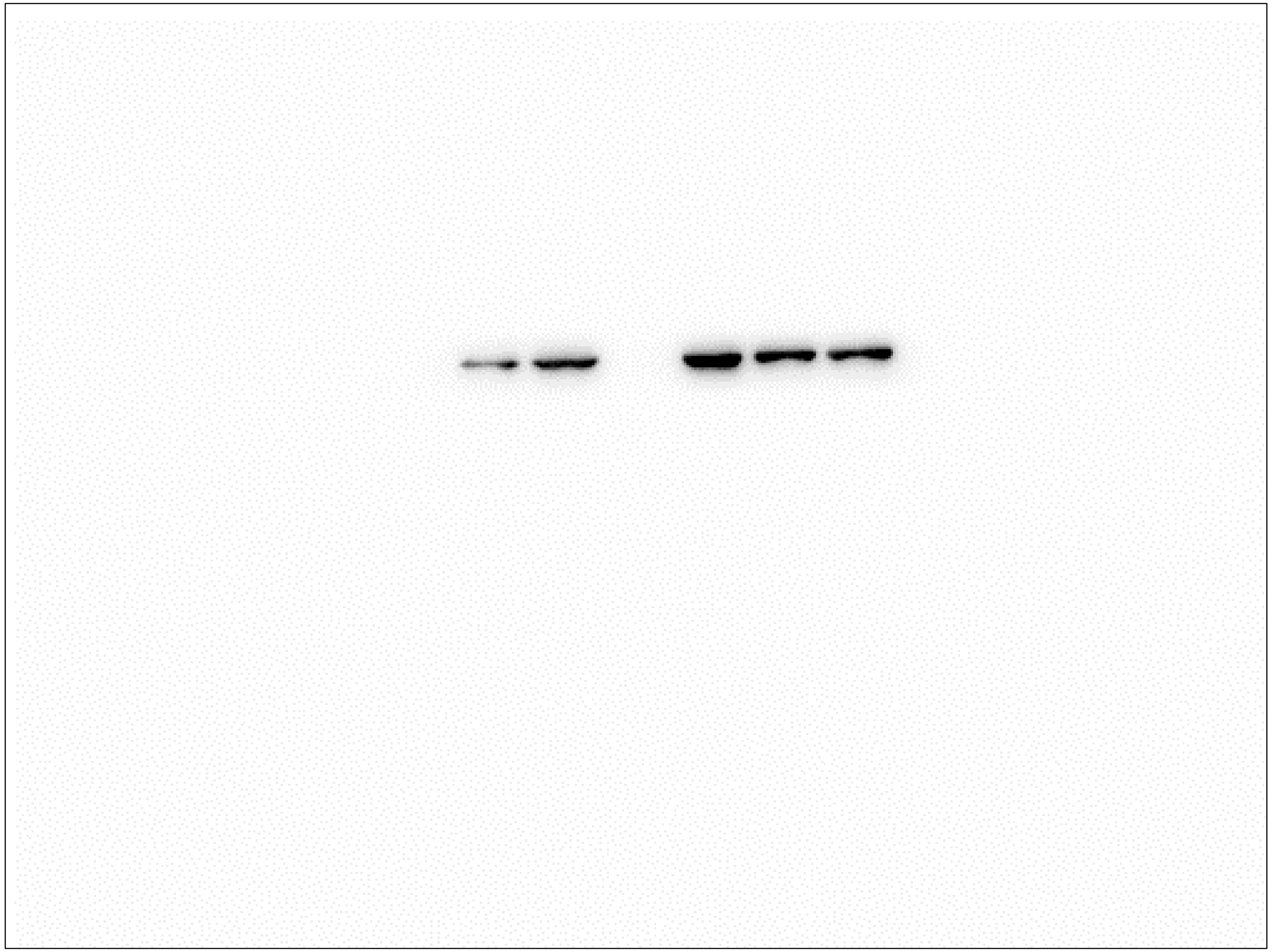

Figure 4D

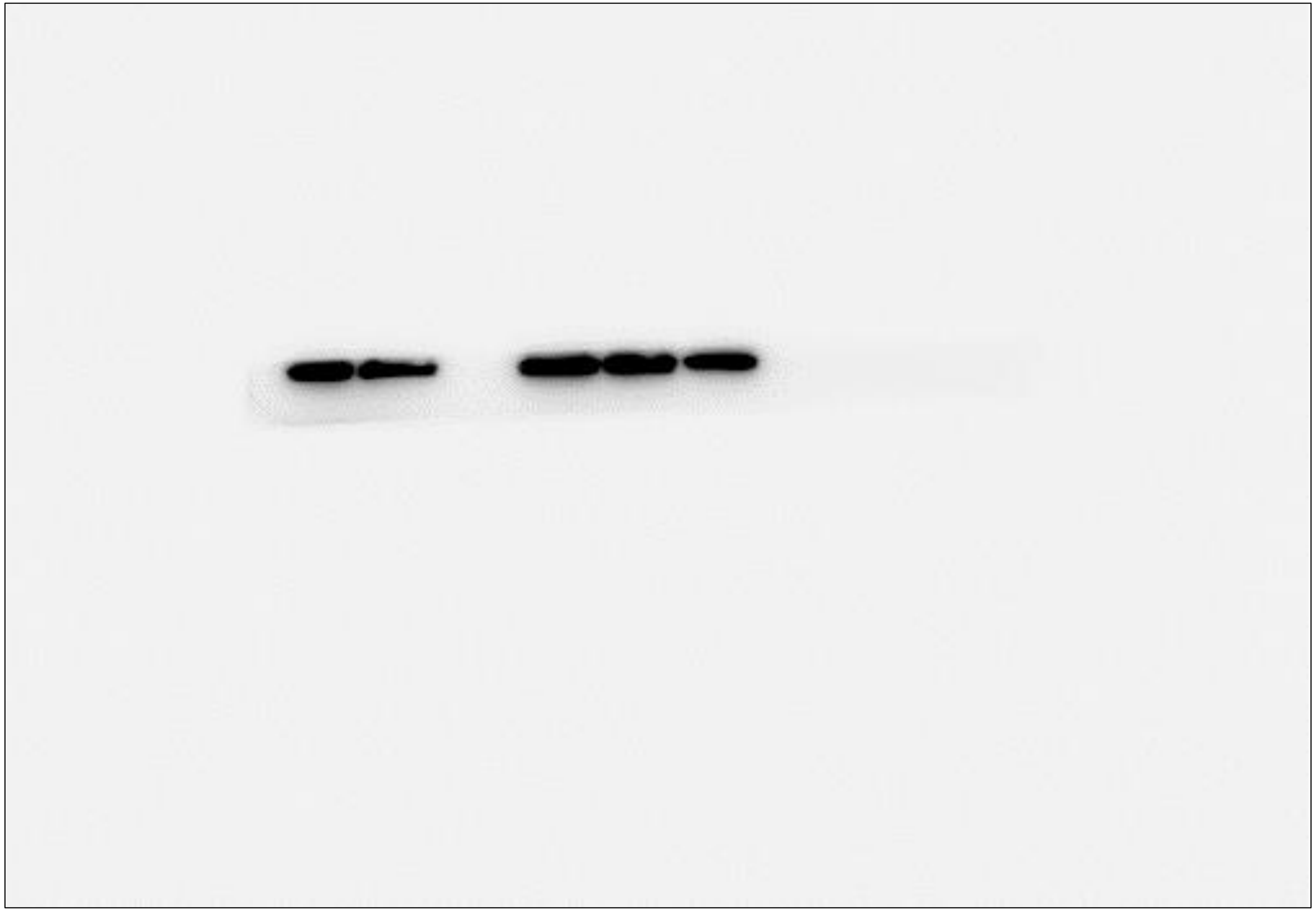

Figure 4D

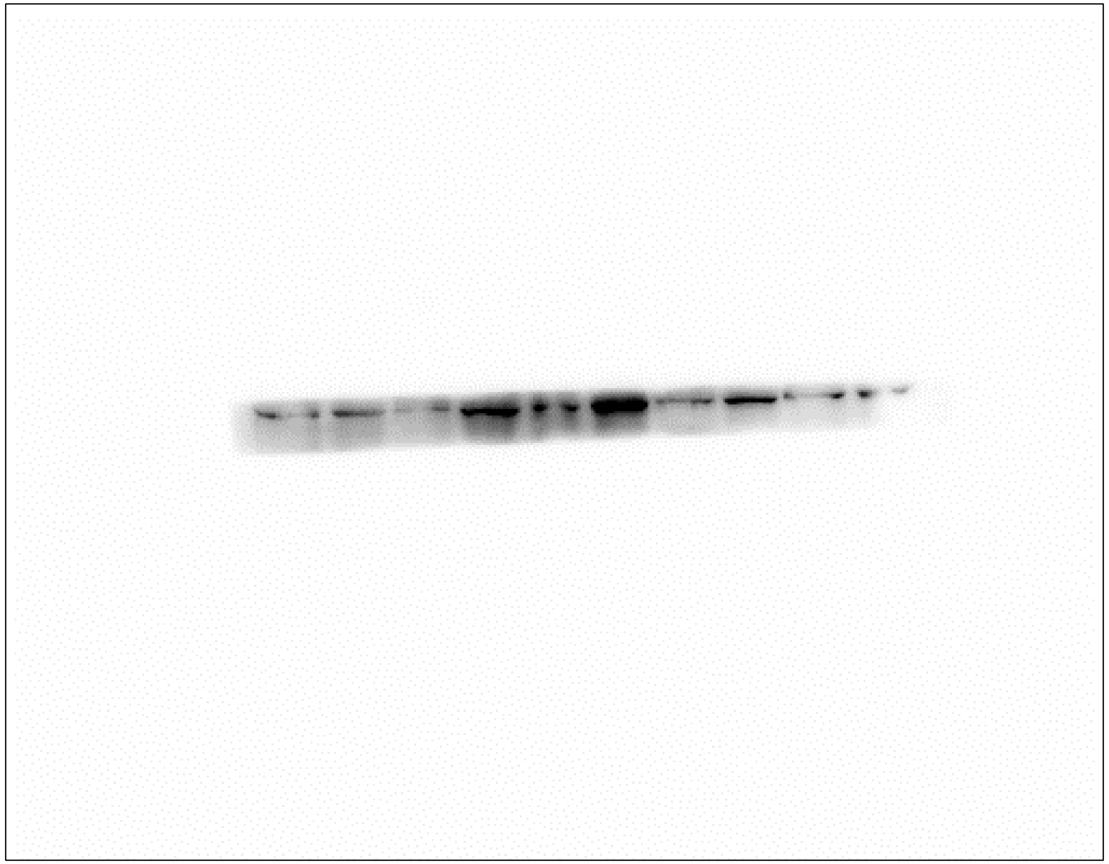

Figure 4D

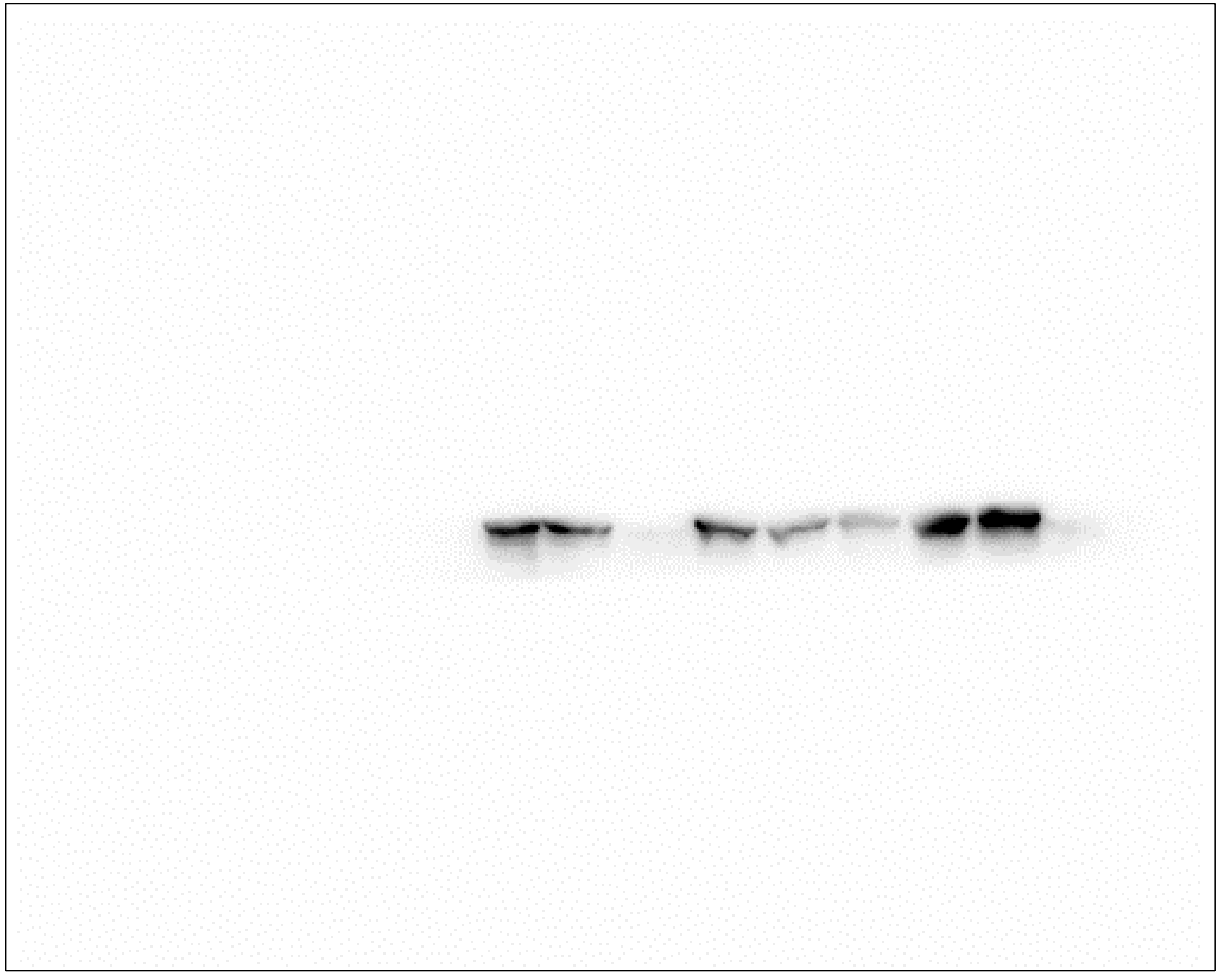

Figure 4D

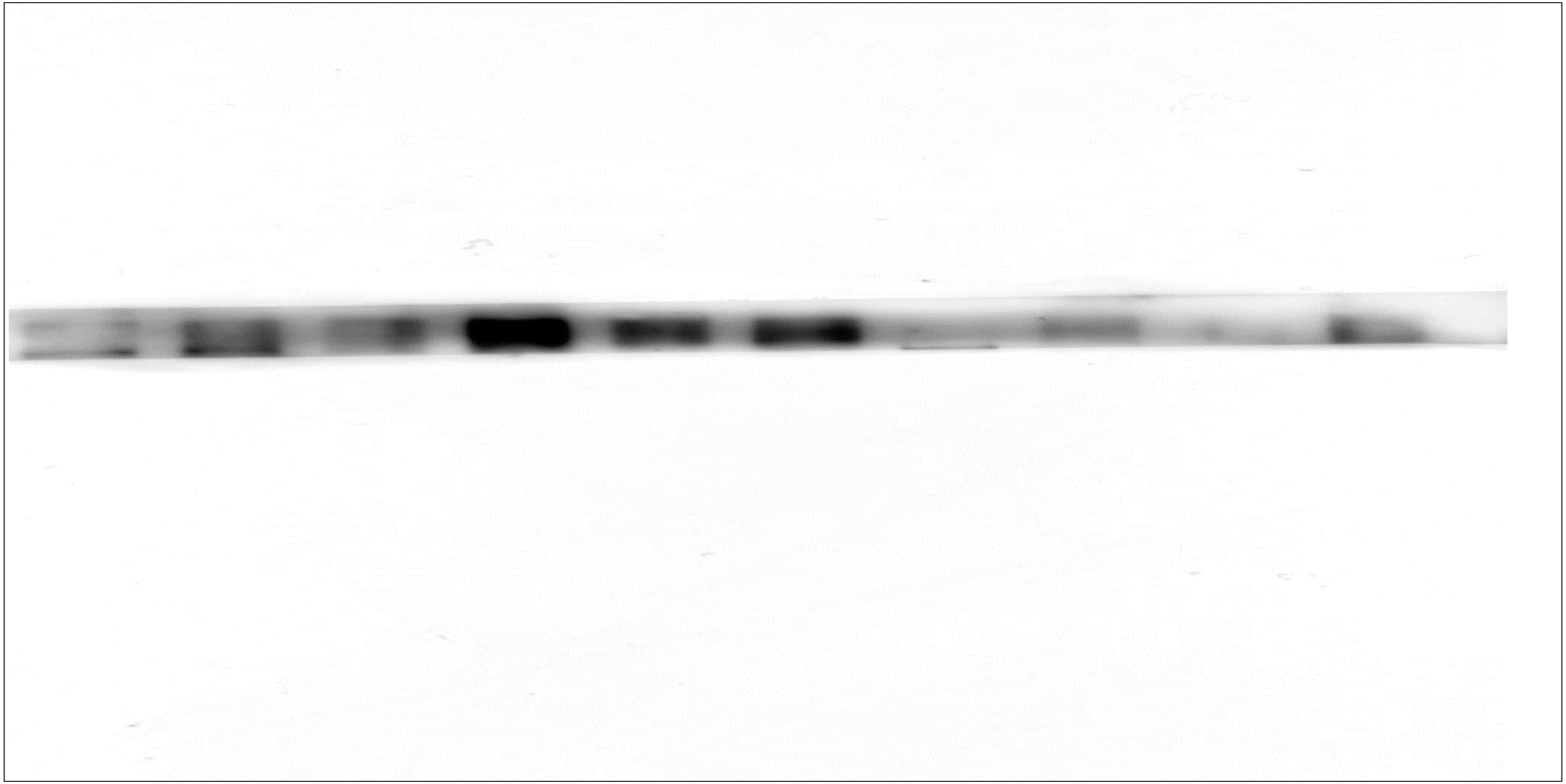

Figure 4D

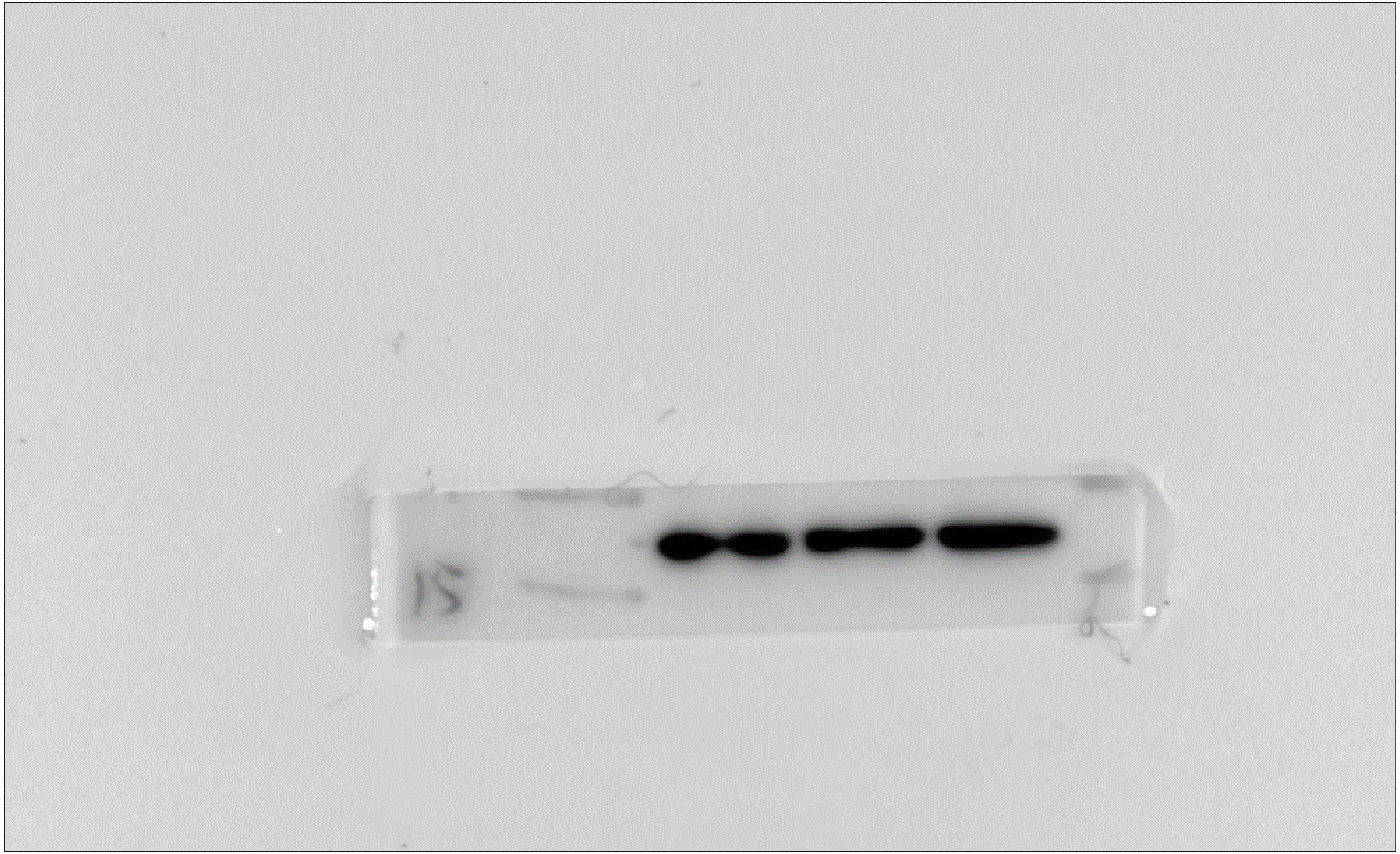

Supplement: Supplementary file 3 — Original Data File [file 41420_2022_1055_MOESM3_ESM.pdf]
